# Identification of *Burkholderia pseudomallei* Genes Induced During Infection of Macrophages by Differential Fluorescence Induction

**DOI:** 10.3389/fmicb.2020.00072

**Published:** 2020-02-21

**Authors:** Siroj Jitprasutwit, Niramol Jitprasutwit, Claudia M. Hemsley, Nattawat Onlamoon, Patoo Withatanung, Veerachat Muangsombut, Paiboon Vattanaviboon, Joanne M. Stevens, Catherine Ong, Mark P. Stevens, Richard W. Titball, Sunee Korbsrisate

**Affiliations:** ^1^Department of Immunology, Faculty of Medicine Siriraj Hospital, Mahidol University, Bangkok, Thailand; ^2^Department of Biosciences, University of Exeter, Exeter, United Kingdom; ^3^Siriraj Research Group in Immunobiology and Therapeutic Sciences, Faculty of Medicine Siriraj Hospital, Mahidol University, Bangkok, Thailand; ^4^Laboratory of Biotechnology, Chulabhorn Research Institute, Bangkok, Thailand; ^5^The Roslin Institute, The Royal (Dick) School of Veterinary Studies, University of Edinburgh, Edinburgh, United Kingdom; ^6^Defence Medical and Environmental Research Institute, DSO National Laboratories, Singapore, Singapore

**Keywords:** *Burkholderia pseudomallei*, intracellular, macrophage, gene expression, promoter trap library, screen, differential fluorescence induction

## Abstract

*Burkholderia pseudomallei*, the causative agent of melioidosis, can survive and replicate in macrophages. Little is known about *B. pseudomallei* genes that are induced during macrophage infection. We constructed a *B. pseudomallei* K96243 promoter trap library with genomic DNA fragments fused to the 5′ end of a plasmid-borne gene encoding enhanced green fluorescent protein (eGFP). Microarray analysis showed that the library spanned 88% of the *B. pseudomallei* genome. The recombinant plasmids were introduced into *Burkholderia thailandensis* E264, and promoter fusions active during *in vitro* culture were removed. J774A.1 murine macrophages were infected with the promoter trap library, and J774A.1 cells containing fluorescent bacteria carrying plasmids with active promoters were isolated using flow cytometric-based cell sorting. Candidate macrophage-induced *B. pseudomallei* genes were identified from the location of the insertions containing an active promoter activity. A proportion of the 138 genes identified in this way have been previously reported to be involved in metabolism and transport, virulence, or adaptation. Novel macrophage-induced *B. pseudomallei* genes were also identified. Quantitative reverse-transcription PCR analysis of 13 selected genes confirmed gene induction during macrophage infection. Deletion mutants of two macrophage-induced genes from this study were attenuated in *Galleria mellonella* larvae, suggesting roles in virulence. *B. pseudomallei* genes activated during macrophage infection may contribute to intracellular life and pathogenesis and merit further investigation toward control strategies for melioidosis.

## Introduction

*Burkholderia pseudomallei* is a saprophytic bacterial pathogen that causes melioidosis, primarily in Southeast Asia and Northern Australia (Wuthiekanun et al., 2005; Wiersinga et al., 2006). However, the global distribution of melioidosis is now believed to be much broader, and it is predicted that there are 45 countries where the disease is underreported and 34 countries where the disease is likely to be present (Limmathurotsakul et al., 2016). Humans often acquire melioidosis through wounds or by the inhalation of contaminated dust or water droplets (Wiersinga et al., 2006). Melioidosis in humans can appear as a rapidly fatal septicemia, acute pneumonia, or subacute disease. *B. pseudomallei* is resistant to many antibiotics, making it difficult to treat, and currently, there is no vaccine to protect against melioidosis (Dance, 2000). In the United States, *B. pseudomallei* is a Tier 1 select agent, owing to its potential to cause a mass casualty event after a deliberate release (Wagar, 2016). Additionally, *B. pseudomallei* is listed in Schedule five pathogens and toxins controlled under the Anti-Terrorism, Crime and Security Act (ATCSA) in the United Kingdom.

*Burkholderia pseudomallei* deploys a variety of virulence factors during its interactions with host cells, and these contribute to immune evasion and pathogenesis (Wiersinga et al., 2006; Galyov et al., 2010). After the bacteria are taken up by phagocytic cells, a Type III secretion system (T3SS-3) mediates the escape of the bacteria from the phagosome (Stevens et al., 2002; Gong et al., 2011). Once they are free in the cytoplasm, actin-based motility allows the bacteria to spread intracellularly and intercellularly (Stevens M.P. et al., 2005) and a Type VI secretion system (T6SS-5) promotes cell fusion, enabling *B. pseudomallei* to form multinucleated giant cells (MNGCs) and spread from cell to cell to evade immune surveillance (Burtnick et al., 2011; Schwarz et al., 2014).

To gain insight into how *B. pseudomallei* survives and to establish the infection in host cells, a range of techniques have been used. A comprehensive DNA microarray has been used to investigate the transcription profile of *B. pseudomallei* within human U937 macrophages (Chieng et al., 2012). RNA sequencing has been also applied to study the transcriptome of *B. pseudomallei* in the RAW264.7 murine macrophage cell line and during acute respiratory infection of inbred mice (Ooi et al., 2013). Transposon mutagenesis has also been used to identify genes required for virulence and the intracellular lifestyle of *B. pseudomallei* (Cuccui et al., 2007; Moule et al., 2015).

*In vivo* expression technology (IVET) has also been used to study bacterial gene expression (Valdivia and Falkow, 1997). IVET is a promoter-trapping technique that reveals bacterial promoters which are active during the interaction with the host by screening for expression of a reporter gene. Previously, an IVET screen using a chloramphenicol resistance gene as the reporter has been employed to identify *B. pseudomallei* promoters activated in RAW264.7 cells. This revealed that a promoter driving the T6SS-5 gene cluster was induced following uptake by macrophages (Shalom et al., 2007). However, when using antibiotic resistance genes as reporters, weakly expressed promoters may not be identified by this methodology because they fail to activate sufficient gene expression to confer phenotypic resistance. This drawback is circumvented by using differential fluorescence induction (DFI), in which fluorescence-activated cell sorting (FACS) is used to isolate host cells harboring fluorescent bacteria that carry active promoter fusions to a fluorescent reporter protein (Bumann and Valdivia, 2007). This technique allows high-throughput screening for positive clones that can be isolated directly from infected cells, or animal tissues, under various conditions.

**FIGURE 1:**
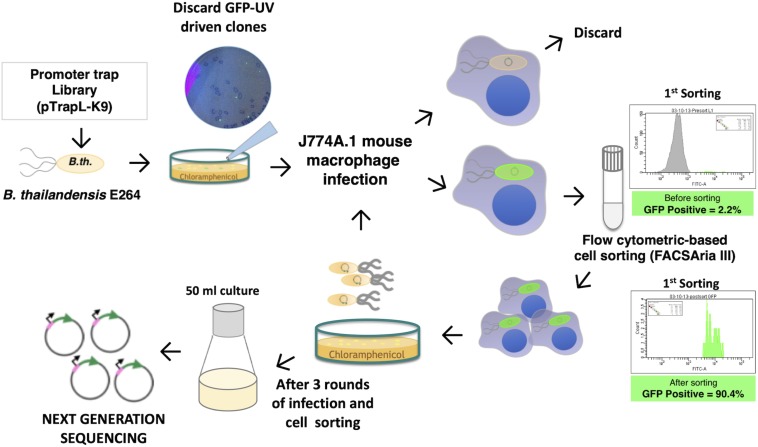
Scheme for identification of macrophage-induced genes of *Burkholderia pseudomallei* K96243 in *Burkholderia thailandensis* E264 using differential fluorescence induction (DFI). DNA fragments of *B. pseudomallei* were ligated into a promoterless-eGFP plasmid, resulting in a pTrapL-K9 library that was then transformed into *B. thailandensis* E264. Clones that fluoresced on the Luria-Bertani (LB) agar medium under UV light were discarded, and only non-fluorescent colonies on LB agar were pooled and subjected to infected J774A.1 cells. Enhanced green fluorescent protein (eGFP)-positive clones were isolated by cell sorting. Intracellular bacteria were released from sorted cells for reinfection. In a total of three rounds of infection, the eGFP-expressing bacteria were cultured for plasmid extraction and sequencing.

In this study, we constructed a promoter library of *B. pseudomallei* using a plasmid containing a promoterless gene encoding enhanced green fluorescent protein (eGFP) and then introduced this into the closely related bacterium *Burkholderia thailandensis* to allow screening of infected macrophages by FACS under biosafety Level 2 conditions. The library was depleted of fusions that were active during growth on laboratory medium. After selection of clones activated during infection of macrophages, DNA sequencing identified *B. pseudomallei* promoters that were induced during infection. A summary of the method used to isolate clones showing differential eGFP expression in macrophages is shown in Figure 1. This identified promoters upstream of genes encoding proteins with a broad range of functions, including virulence-associated proteins and proteins which have not previously been shown to play roles in *B. pseudomallei*–host cell interactions. These gene products can now be evaluated as targets for the development of vaccines or novel therapeutics.

## Materials and Methods

### Bacterial Strains, Mouse Macrophages, and Culture Conditions

*Burkholderia pseudomallei* strain K96243 was isolated from a human melioidosis patient (Holden et al., 2004). *B. thailandensis* E264 has been genome sequenced (Yu et al., 2006) and is widely used as a surrogate for *B. pseudomallei* in cell-based assays and has been confirmed to deploy many of the same virulence factors during interaction with macrophages. Bacterial stocks were kept in 15% (v/v) glycerol at −70°C. The bacteria were cultured in Luria-Bertani (LB) broth or LB agar (Criterion) with or without 50 μg/ml of chloramphenicol (Sigma). Bacteria were grown at 37°C. All experiments working with *B. pseudomallei* were conducted at Mahidol University in a biosafety level 3 laboratory with approval by the Technical Biosafety Committee of National Center for Genetic Engineering and Biotechnology (BIOTEC). Genetic modification for the introduction of *B. pseudomallei* DNA fragments (700–1,500 bp) into *B. thailandensis* was approved by the Siriraj Biosafety Risk Management Taskforce (Approval No. SI2017-007).

The J774A.1 mouse macrophage cell line was obtained from the American Type Culture Collection (ATCC). The cells were routinely grown and maintained in Dulbecco’s modified Eagle medium (DMEM; Gibco), supplemented with 10% heat-inactivated (30 min, 56°C) fetal bovine serum (FBS; HyClone).

### Construction of Promoterless-eGFP Plasmid Vector

The pBHR4-*gfp* plasmid was modified from a variant of broad-host-range plasmid pBHR4-*groS*-RFP (Wand et al., 2011) where the gene encoding red fluorescent protein (RFP) had been replaced with one encoding eGFP. The eGFP gene was amplified by PCR using a forward primer (Trap-F) that anneals to the start of the eGFP gene with added restriction sites for *SacI*, *BglII*, and *SpeI* and a reverse primer (Trap-R) that anneals to the end of the eGFP gene with an added *Bam*HI restriction site. The constitutive *groS* promoter (*PgroS*) and *rfp* of the original pBHR4-*groS*-RFP plasmid was removed by restriction enzyme digestion using *Sac*I and *Bam*HI, which resulted in the pBHR4-backbone fragment and a smaller *groS*-RFP fragment. The linearized backbone was then ligated together with *Sac*I and *Bam*HI restricted eGFP gene fragment PCR products to create the promoterless-eGFP plasmid pBHR4-*gfp*. A constitutive eGFP expression plasmid (pBHR4-*groS*-GFP) was constructed for use as a positive control when macrophages were infected with eGFP-expressing bacteria. The *groS* promoter was removed from the original pBHR4-*groS*-RFP plasmid by restriction enzyme digestion using *Sac*I and *Spe*I. The resulting *groS* promoter fragment was then cloned into *Sac*I/*Spe*I restricted plasmid pBHR4-*gfp* to create plasmid pBHR4-*groS*-eGFP.

### *B. pseudomallei* Genomic DNA Preparation

A single colony of *B. pseudomallei* K96243 was inoculated into 10 ml of LB broth and cultured with shaking for 16 h. Two milliliters of the culture was transferred to a microcentrifuge tube and centrifuged at 14,000 × g for 1 min. DNA was extracted from the pelleted cells using a DNA extraction step according to the manufacturer’s instructions (Geneaid Biotech). The DNA pellet was resuspended in an elution buffer and stored at −20°C until used. The yield and purity of the DNA were determined by spectrophotometry (NanoDrop Technologies).

### Promoter Trap Library Construction

To construct the promoter trap library, *B. pseudomallei* K96243 genomic DNA was partially digested with the restriction enzyme *Sau*3AI. After agarose gel electrophoresis, DNA fragments in the range of 700–1,500 bp were eluted from the gel. The purified DNA fragments were ligated to dephosphorylated *Bgl*II-digested plasmid pBHR4-*gfp*5′ of the promoterless-eGFP gene. The resulting library of cloned *B. pseudomallei* K96243 genomic fragments (pTrapL-K9) was introduced into *B. thailandensis* E264 by electroporation. The recombinant clones (approximately 40,000 colonies) were selected on LB agar supplemented with 50 μg/ml of chloramphenicol and pooled. An aliquot of the *B. thailandensis* E264 pTrapL-K9 library was subcultured in LB broth supplemented with chloramphenicol for plasmid extraction. To assess library coverage, the extracted plasmids were labeled with Cy3 (green fluorescent dye) and hybridized to a high-density tiling microarray based on the *B. pseudomallei* K96243 genome, essentially as described (Jitprasutwit et al., 2014). The array comprises 384,926 50-mer probes representing both sense and antisense strands at an average resolution of 35 bp (with a mean overlap between probes of 15 bp) and represents 95.1% of the *B. pseudomallei* K96243 genome, including intergenic regions (Jitprasutwit et al., 2014). To identify *B. pseudomallei* genes that were preferentially induced in macrophages, *B. thailandensis* E264 clones from the pTrapL-K9 library with promoters active during culture on an LB agar medium (based on green fluorescence under UV light) were discarded. Only non-fluorescent colonies on LB agar were pooled and used to infect J774A.1 cells.

### J774A.1 Mouse Macrophage Infection and Cell Sorting

J774A.1 cell monolayers in 75-cm^2^ tissue culture flasks (Costar) were infected with either *B. pseudomallei* or *B. thailandensis*. Various multiplicities of infection (MOIs) were tested to determine the optimal MOI for screening of the promoter trap library. J774A.1 macrophages were infected with an overnight culture of *B. thailandensis* E264 carrying the pTrapL-K9 library using MOIs of 15, 20, and 25 and incubated at 37°C under 5% CO_2_ atmosphere for 1 h to bring bacteria into contact with the cells and allow bacterial entry. Cell monolayers were washed thrice with pre-warmed phosphate-buffered saline (PBS) to remove the non-adherent bacteria and overlaid with medium containing 1 ml of 250 μg/ml of kanamycin (Gibco) to kill extracellular bacteria for 2 h. At 3 h post infection, the monolayers were washed with pre-warmed PBS to remove antibiotic and overlaid with DMEM. The monolayers were further incubated for 3 h. After 6 h post infection, the monolayers were washed with pre-warmed PBS. The infected cells were treated with 0.05% trypsin–EDTA (Gibco), washed with PBS, and subjected to cell sorting. Briefly, the infected cells were suspended in PBS containing 2% FBS and immediately transferred into sample tubes that were compatible for the BD FACSAria III cell sorter (BD Biosciences). A doublet discrimination gating strategy was applied to identify a population of singlet cells. Fluorescence due to eGFP was detected using the channel for fluorescein isothiocyanate (FITC). Fluorescence intensity from uninfected macrophages was used as a cutoff for fluorescence background. A sorted population inferred to comprise eGFP-positive cells was identified on a two-dimensional dot plot presenting forward-scatter signal versus FITC fluorescent signal. The eGFP-positive cells were sorted into a collection tube containing PBS with 2% FBS and used for DFI screening. After cell sorting, intracellular bacteria were released using 0.1% Triton X-100 (Sigma) in PBS to lyse the sorted cells. These bacteria were then used to repeat the infection and screening process, with a total of three rounds of macrophage infection prior to recovery of bacterial clones for sequence analysis.

### Plasmid DNA Extraction and Sequencing

Plasmid DNA was extracted from *B. thailandensis* eGFP-positive clones after three passages in macrophages using the plasmid DNA isolation kit (Geneaid Biotech). In brief, a pool of *B. thailandensis* was inoculated into 50 ml of LB broth supplemented with 50 μg/ml chloramphenicol and cultured at 37°C with shaking for 16 h. The plasmid DNA was extracted according to the manufacturer’s instructions and the yield and purity was determined using a spectrophotometer (NanoDrop Technologies). DNA inserts in the sorted pTrapL-K9 clones were amplified by PCR using primers Trap-F and Trap-R (Supplementary Table 1), which anneal to the plasmid backbone flanking the inserts. The amplified PCR products were used directly as input for Illumina sequencing. Sequencing libraries were prepared using a NEB Next DNA library preparation kit according to the manufacturer’s instructions (New England Biolabs). Sequencing was performed on an Illumina MiSeq flow cell generating 250-bp paired end reads. Illumina adapters were removed and sequences quality trimmed using ea-utils (Aronesty, 2010). The resulting reads were remapped onto the concatenated chromosomes I and II of *B. pseudomallei* K96243 sequence using the BWA aligner (Li and Durbin, 2009). The position of the mapped reads were extracted from the bam files, and reads were visualized using the Artemis genome viewer (Rutherford et al., 2000).

### Bacterial RNA Extraction and cDNA Synthesis

To validate candidate *in vivo* induced promoters from the library screen, gene expression of *B. pseudomallei* K96243 during macrophage infection was compared with bacteria grown in cell culture media. An overnight culture of *B. pseudomallei* was diluted in DMEM supplemented with 10% FBS to give an MOI of 20. This bacterial suspension was used to infect J774A.1 macrophages. Infected macrophages were incubated at 37°C under 5% CO_2_ atmosphere for 1 h. As above, the extracellular bacteria were removed by washing and killed by adding of 250 μg/ml of kanamycin. In parallel, the same bacterial suspension was grown in DMEM supplemented with 10% FBS and incubated at 37°C under 5% CO_2_ atmosphere. At each time point (2 and 4 h post infection), bacterial RNA was extracted from infected J774A.1 cells by a differential lysis method with modifications (Chieng et al., 2012). Macrophage monolayers were lysed in 0.1% Triton X-100 in PBS (Sigma) for 5 min at 37°C. Lysates were collected and subjected to differential centrifugation; first at 800 × *g* for 5 min to sediment macrophage cells and cellular debris, and secondly at 8,000 × *g* for 10 min to pellet bacterial cells. Similarly, the bacteria in DMEM supplemented with 10% FBS were harvested by centrifugation at the same time points. Pellets were immediately subjected to RNA extraction using the Total RNA Mini Kit according to the manufacturer’s instructions (Geneaid). To remove traces of genomic DNA, the RNA samples were treated with DNase I (Promega). SuperScript III First-Strand Synthesis System (Invitrogen) was used to convert total RNA into cDNA according to the manufacturer’s instructions. The yield and purity of the RNA was determined by spectrophotometry (NanoDrop Technologies). The absence of DNA contamination was confirmed by PCR using primers specific to 23S ribosomal RNA genes (23S rRNA) of *B. pseudomallei* before proceeding to cDNA synthesis.

### Quantitative Real-Time PCR

Quantitative real-time PCR (qRT-PCR) was performed on 50-ng cDNA in a final volume of 20 μl of LightCycler^®^ FastStart DNA Master PLUS SYBR Green I (Roche). All experiments were conducted with three biological replicates and relative expression ratios were calculated by comparing the *C*_*t*_ value of the *in vivo* condition (infected macrophages) to the *in vitro* control (growth in cell culture medium). The genes coding for 23S rRNA and cytochrome d ubiquinol oxidase subunit II (*cydB*) were used as the reference genes for normalization. These genes were chosen as references because their expression does not differ significantly between culture *in vitro* and in infected macrophages (Chieng et al., 2012). The primers for amplification of specific genes are described in Supplementary Table 2. The Livak method (Livak and Schmittgen, 2001) was used to determine the relative expression of selected genes in macrophages (*in vivo*) compared to during growth in cell culture medium. After normalization, the expression of each selected gene against the reference gene compensates for any difference in the amount of cDNA per sample, and a differential gene expression value was calculated from the fold change of gene expression in *B. pseudomallei* infecting macrophages relative to those grown in cell culture medium at the specified time points.

### Construction of *B. pseudomallei* Mutants

Deletion mutants were generated using a positive-selection suicide replicon as described in a previous study, with some modifications (Logue et al., 2009). Briefly, primers were designed to amplify 400-bp upstream and downstream regions flanking the target genes, *bpss1622* and *bpss2104*. The upstream and downstream amplicons were ligated using *Apa*I and *Xba*I restriction sites for *bpss1622* and *bpss2104*, respectively. The amplicons fused in this way were ligated with pJCB12 suicide vectors at *Spe*I and *Xba*I restriction sites for *Δbpss1622* or *Spe*I and *Sph*I restriction sites for *Δbpss2104* and then transformed into *Escherichia coli* MFD *pir*^+^. Next, the donor *E. coli* harboring pJCB12Δ*bpss1622* or pJCB12Δ*bpss2104* were conjugated into *B. pseudomallei* K96243 by filter mating (Smith and Guild, 1980). Resulting *B. pseudomallei* merodiploid strains were selected on LB agar supplemented with 50 μg/ml of chloramphenicol and 100 μg/ml of diaminopimelic acid (Sigma). The selected positive merodiploid strains were subcultured in the absence of chloramphenicol and plated on LB agar lacking NaCl and containing 20% (w/v) sucrose and incubated at room temperature to select double recombinants in which the pJCB12 replicons had excised from the chromosome. Candidate mutants were identified on the basis of sucrose resistance and sensitivity to chloramphenicol. Deletion of the target genes in strains that had undergone successful allelic exchange was validated by PCR.

### Virulence in *Galleria mellonella*

Groups of 10 *G. mellonella* larvae were used for virulence studies. Overnight cultures of *B. pseudomallei* wild type, Δ*bpss1622*, or Δ*bpss2104* mutants were diluted in PBS. Groups of larvae were challenged by injection of an approximately 1000 colony-forming unit (CFU) of each strain at the posterior proleg and incubated in the dark at 37°C (Harding et al., 2013). Another group of larvae were injected with 10 μl of PBS as a control. At intervals between 24 and 60 h after injection, the larvae were scored for death, evidenced as no response to gentle pushing with a pipette tip, and any color change to brown or black was recorded periodically. This experiment was performed in triplicate.

### Statistical Analysis

Data from three independent experiments were collected and analyzed using the student’s unpaired *t*-test using the GraphPad Prism 7 software. For *G. mellonella* virulence studies, a log-rank (Mantel–Cox) test was used to compare survival curves using the GraphPad Prism 7 software. A *P*-value of <0.05 was considered significant.

## Results

### Construction of a *B. pseudomallei* K96243 Promoter Trap Library

**FIGURE 2:**
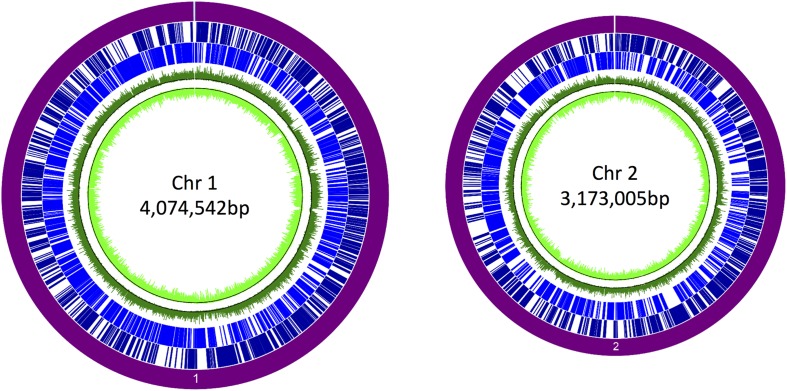
Coverage of the promoter trap library in *Burkholderia pseudomallei* K96243. The purple circle denotes each chromosome. Dark blue lines denote genes on the forward strand, and light blue lines denote genes on the reverse strand. The corresponding signals obtained by hybridization of the pTrapL-K9 library to the tiling array are indicated by the green circles. The black line on the green tracks is the cutoff for background. Percentage coverage of the genome by the pTrapL-K9 library (88%) has been corrected for the fact that the microarray probes cover 95.1% of the *B. pseudomallei* genome.

We first constructed a library of size-selected (700–1,500 bp) DNA fragments of *B. pseudomallei*, which were ligated into a broad-host-range vector upstream of a promoterless gene encoding eGFP. The resulting pTrapL-K9 library was transformed into *B. thailandensis* E264 and approximately 40,000 colonies were obtained. To test the library was adequately diverse and comprehensively spanned the *B. pseudomallei* genome, plasmid DNA extracted from the complete library was labeled with Cy3 fluorescent dye and hybridized to a high-density *B. pseudomallei* K96243 tiling microarray. Analysis of the hybridization pattern indicated that 88% of *B. pseudomallei* K96243 genome was represented in the plasmid library with good coverage across both chromosomes (Figure 2).

### Isolation of Clones Showing Differential eGFP Expression in Macrophages

Following depletion of clones expressing eGFP during culture on LB agar, J774A.1 murine macrophages were infected with the remaining *B. thailandensis* E264 pTrapL-K9 library using an optimal MOI. Internalization efficiencies of *B. thailandensis* E264 were increased when using a higher MOI and the number of bacteria recovered from infected macrophages was significantly lower (*P* < 0.05) when using an MOI of 15, compared to using an MOI of 20 or 25. However, there was no significant difference between the numbers of bacteria recovered from macrophages after infection at an MOI of 20 or 25 (Supplementary Figure 1). Therefore, for subsequent library screening, J774A.1 cells were infected with the *B. thailandensis* E264 pTrapL-K9 library at an MOI of 20. With this inoculum, 5.97 × 10^6^ CFU were recovered from approximately 8 × 10^6^ infected cells, indicating that on average there were 0.7 bacteria per cell at the point of analysis.

**FIGURE 3:**
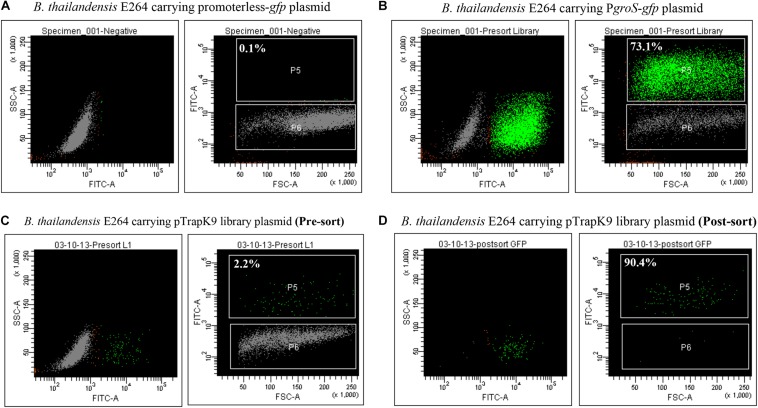
Enhanced green fluorescent protein (eGFP) expression in J774A.1 macrophages. **(A)** Gating for eGFP-positive cells was set according to the fluorescent background of macrophages infected with *Burkholderia thailandensis* carrying a promoterless-*gfp* control plasmid. **(B)** eGFP expression was observed in macrophages infected with *B. thailandensis* carrying *PgroS*-eGFP. **(C)** Macrophages infected with the *B. thailandensis* promoter trap library (pre-sorted). **(D)** The post-sorted macrophage infected with *B. thailandensis* promoter trap library. The percentages of eGFP-positive cells are indicated.

J774A.1 cells infected with the *B. thailandensis* E264 pTrapL-K9 library were screened for a fluorescent signal at 6 h after macrophage infection. The rationale for screening the library at 6 h post infection was that we could maximize the number of cells that were infected but before cell-cell fusion occurred (Wand et al., 2011). The time is sufficient for internalized bacteria to escape phagosomes and display actin-based motility (Stevens J.M. et al., 2005; Wand et al., 2011). A sequential gating strategy was used to minimize false positives. Macrophages infected with *B. thailandensis* carrying the promoterless-eGFP plasmid were included as a negative control. Only 0.19% of the macrophage population exhibited a dim-auto-fluorescence signal from uninfected macrophages. This data was used to determine the background fluorescence intensity for further analysis (Figure 3A, Supplementary Figure 2). *B. thailandensis* harboring pBHR4-*groS*-eGFP was used as a positive control for eGFP-positive cells (Figure 3B, Supplementary Figure 2). Levels of fluorescence of these controls were measured in order to set the appropriate gates to distinguish uninfected cells and GFP-negative cells from the fluorescent macrophages inferred to be infected with *B. thailandensis* carrying *in vivo* induced promoters.

In the first round, macrophages infected with the *B. thailandensis* pTrapL-K9 library were sorted and 2.2% of the infected cells were eGFP positive (Figure 3C, Supplementary Figure 2). The sorted eGFP-positive cells were reanalyzed by flow cytometry and 90% of the collected cells were eGFP positive (Figure 3D, Supplementary Figure 2). To further enrich eGFP-positive clones, bacteria were released from the sorted cells by gentle lysis and used to re-infect J774A.1 macrophages. In total, we carried out three rounds of macrophage passage of the library. Finally, bacteria were recovered and plated onto LB agar. Each colony was visualized under a UV light illuminator to confirm that they did not fluoresce when cultured *in vitro* and the resulting colony exhibiting green fluorescence was not detected (data not shown). These clones were collected and stored.

### Bacterial Virulence and Oxidative Stress Response Genes Were Induced in Infected Macrophages

**FIGURE 4:**
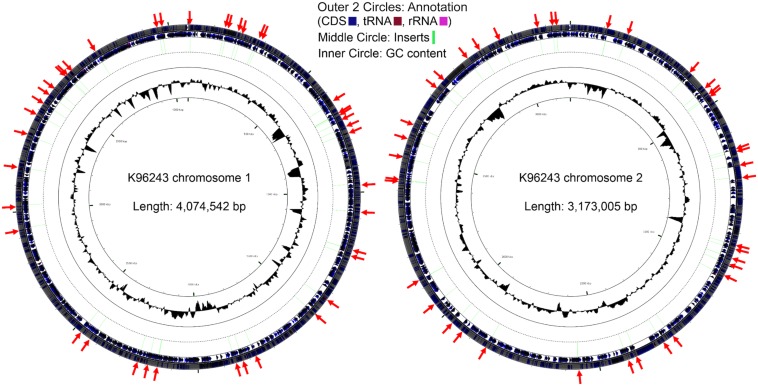
Distribution of the *Burkholderia pseudomallei* genes located downstream of the identified putative inducible promoters during macrophage infection. Locations of the genes immediately downstream of the identified macrophage putative promoters (red arrows) across the respective chromosomes.

Colonies of the bacteria that fluoresced in macrophages but not on agar were pooled and plasmids extracted. Amplicons for the inserts in the recovered pTrapL-K9 plasmids were sequenced by Illumina MiSeq analysis. In total 552,458 reads were obtained, of which 503,624 reads (91.16%) mapped onto the *B. pseudomallei* K96243 reference genome and this revealed 138 different regions distributed across the *B. pseudomallei* K96243 genome, with 60% mapped to chromosome 2 (Figure 4). The length of Illumina mapped regions ranged from 33 to 567 bp (134 bp on average). Within the 138 regions identified, 2 to 8,452 sequence reads mapped to the *B. pseudomallei* K96243 genome. The locations of mapped regions within the genome were visualized using Artemis and genes at the 3′ end of the cloned fragments were identified. The *B. pseudomallei* genes associated with macrophage-induced promoters were categorized into 2 groups, according to the distance between the 5′ end of the mapped regions and the predicted start codon of the associated gene. Mapped regions upstream of the associated gene or overlapping with the predicted start codon (*n* = 27) were assigned to Group A and mapped regions within a predicted coding sequence (*n* = 111) were assigned to Group B (Supplementary Table 2). The possibility that the genes associated with the mapped fragment were part of an operon was assessed from previously reported condition-dependent transcriptome data for *B. pseudomallei* (Ooi et al., 2013). We found that 33% of genes associated with mapped regions were not part of an operon, 22% were the first gene in a predicted operon, 17% were the last gene of the predicted operon and 28% of associated genes were located at another position within the operon. The proportion of promoters identified that are predicted to control transcription of single genes is broadly consistent with the predicted number of monocistronic transcripts from sequencing of the genome (Holden et al., 2004) and whole-genome transcriptome profiling (Ooi et al., 2013).

The functions of genes 3′ end of cloned fragments inferred to contain macrophage-induced promoters were predicted from annotation in GenBank and EMBL or published literature. Known virulence factors were identified, including flagellin (BPSL3319), the T3SS effector protein BapC (BPSS1526), the adhesin BoaA (BPSS0796), and catalase-peroxidase KatG that is predicted to be involved in resistance to oxidative stress (BPSL2865). Moreover, we identified predicted regulatory systems as macrophage-induced, including the *trans-*membrane invasion-related two-component sensor IrlS protein (BPSS1039) and a metal-related two-component system IrlS2 (BPSS1995). Some of the genes we identified are predicted to be involved in responding to environmental signals inside macrophages (Supplementary Table 3). Additionally, the genes we identified that are involved in LPS biosynthesis and RNA modification have been reported to be upregulated in *B. thailandensis* grown in anoxic conditions (Peano et al., 2014).

We compared our gene list with previously reported datasets, for example from mutagenesis with *in vivo* negative selection (Moule et al., 2015), the transcriptome under oxidative stress (Jitprasutwit et al., 2014), *in silico* prediction of virulence genes (Schell et al., 2008), and screening for serodiagnostic antigens (Felgner et al., 2009). We found that nearly one third of the genes identified from our promoter trap study had not previously been reported to be associated with virulence or as *in vivo*-induced antigens (Supplementary Table 3).

### Validation of the Putative Macrophage-Induced *B. pseudomallei* Genes by RT-PCR and Virulence Study

As the study used *B. thailandensis* as a surrogate host for screening of the K96243 promoter trap library owing to constraints on cell sorting at biosafety Level 3, we sought to verify that candidate macrophage-induced genes are differentially transcribed during *B. pseudomallei* infection of J774A.1 cells. Additionally, the virulence of selected mutants was tested in *G. mellonella* larvae. A total of 15 genes were selected for validation by qRT-PCR or virulence in *G. mellonella* (Table 1).

**TABLE 1:** *Burkholderia pseudomallei* genes located downstream of putative macrophage-induced promoters and selected for validation using qRT-PCR and mutagenesis.

**No.**	**Gene**	**Strand**	**Position/total genes in operon^a^**	**Number of reads**	**Length of mapped region (bp)**	**Reads mapped within CDS (bp)**	**Gene length (bp)**	**% Coverage**	**Distance (bp)^b^**
**Group A**									
1	bpsl0007*	+	1/3	31	182	122	2,274	5.4%	−60
2	bpsl0125*	+	1/4	31	229	222	1,410	15.5%	−7
3	bpsl0346*	−	1/1	76	96	42	903	4.7%	−54
4	bpsl1534*	+	1/2	27	293	276	1,806	15.3%	−17
5	bpsl2987*	+	6/6	121	342	257	504	50.9%	−85
6	bpss0479*	+	1/1	14	195	51	2,535	2.0%	−144
7	bpss0769*	–	1/2	273	175	60	981	6.1%	−115
8	bpss1442*	+	2/3	1,362	257	109	1,158	9.4%	−150
9	bpss1835*	+	1/1	4	224	171	1,461	11.7%	−53
**Group B**									
10	bpsl3338*	–	1/1	52	191	191	1,818	10.5%	20
11	bpss0547*	+	1/2	121	94	94	1,290	7.3%	987
12	bpss1039*	−	5/5	9	223	223	1,395	15.9%	2
13	bpss1268*	−	3/5	1,666	90	90	1,290	6.9%	3
14	*bpss1622*^#^	+	1/8	3,699	59	66	582	11.3%	56
15	*bpss2104*^#^	–	4/5	537	151	153	3,630	4.2%	1532

^*a*^Ooi et al. (2013). ^*b*^Distance from the 5′ end of the mapped regions to the annotated start codon; a negative value indicates that the start of the mapped region was upstream of the translational start site (Group A); a positive value indicates that the start of the mapped region was downstream of the translational start site, e.g., lying within the CDS (Group B). *The genes were validated by RT-PCR. #The genes were implicated in virulence in *Galleria* by mutagenesis.

Transcription of 13 genes downstream of putative macrophage-induced promoters (nine in Group A and four in Group B) was examined 2 or 4 h after infection of J774A.1 macrophages by *B. pseudomallei* K96243 at an MOI of 20 relative to expression in LB broth after 2 or 4 h of culture by qRT-PCR. *B. pseudomallei* RNA was collected at 2 or 4 h rather than 6 h post infection with *B. thailandensis*, because of the faster growth of *B. pseudomallei* K96243 in macrophages, compared with *B. thailandensis* E264 (Wand et al., 2011; Kovacs-Simon et al., 2019). Fold changes in gene expression were calculated by normalizing the level of cDNA with a reference gene (23S rRNA or *cydB*) prior to comparison of gene expression in macrophages against *in vitro* growth (Table 2). We selected genes from Group A and Group B by choosing from their gene organization. Genes that were not a part of an operon, or were the first, middle, or last gene of a predicted operon, were selected randomly. We included *bpss1498*, encoding a Type VI secretion system protein, as a positive control as the gene is known to be upregulated in macrophages (Chieng et al., 2012).

**TABLE 2:** Expression of 13 genes measured using qRT-PCR in *Burkholderia pseudomallei* during infection of J774A.1 macrophages.

**Gene**	**Description**	**Fold change (log 2 transformed) normalized with**			
		
		***23S rRNA***		***cydB***	
		**2 h**	**4 h**	**2 h**	**4 h**
**Group A^a^**					
*bpsl0007*	Type II secretory pathway	5.84	5.66	2.90	4.30
*bpsl0125*	tRNA and rRNA cytosine-C5-methylases	8.01	9.96	5.07	8.60
*bpsl0346*	Dihydrodipicolinate synthetase	8.56	6.98	5.62	5.62
*bpsl1534*	Poly-beta-hydroxybutyrate polymerase	–1.06	0.64	–4.00	–0.72
*bpsl2987*	Thiol peroxidase	–1.50	0.14	–4.44	–1.22
*bpss0479*	Ribonucleotide reductase	2.61	5.98	–0.33	4.62
*bpss0769*	Esterase/lipase	9.86	12.16	6.92	10.80
*bpss1442*	Hypothetical protein	7.54	7.13	4.60	5.77
*bpss1835*	LPS biosynthesis mannose-1-phosphate guanylyltransferase	5.74	7.18	2.80	5.82
**Group B^b^**					
*bpsl3338*	Methyl-accepting chemotaxis protein	9.38	8.52	6.44	7.16
*bpss0547*	Serine hydroxymethyl transferase	7.04	9.80	4.10	8.44
*bpss1039*	*irls*, two-component response regulator	7.00	6.26	4.06	4.90
*bpss1268*	Arabinose efflux permease	9.06	10.59	6.12	9.23
**Positive control**					
bpss1498*	Type VI secretion system protein TssD-5	6.82	8.69	3.88	7.33

Fold change shown is in comparison with expression in Luria-Bertani (LB) broth. ^a^The start of the mapped regions of the identified genes in this group was upstream of the translational start site. ^b^The start of the mapped regions of the identified genes in this group resided within the CDS. *Upregulated genes in human macrophage-like U937 cells (Chieng et al., 2012).

As expected, we also found increased expression of *bpss1498* after infection of J774A.1 cells with *B. pseudomallei*. Of the 13 genes identified from our promoter trap study, 11 showed greater than twofold increases in expression in bacteria isolated from macrophages compared to bacteria cultured in LB broth. Two genes in Group A, *bpsl1534* and *bpsl2987* encoding poly-beta-hydroxybutyrate polymerase and thiol peroxidase, respectively, showed reduced expression in macrophages compared to LB depending on the time and reference gene used, albeit the magnitude of the change was limited and the direction of the effect was inconsistent. The data obtained for *bpsl1534* and *bpsl2987* by qRT-PCR were not consistent with specific induction of transcription of the genes suggested by the fluorescence-based screen. This may reflect the different sampling times (qRT-PCR at 2 and 4 h post infection vs. 6 h post infection for the DFI screen). Also, it may be that these genes were expressed transiently or that the encoding mRNAs had atypical half-lives.

**FIGURE 5:**
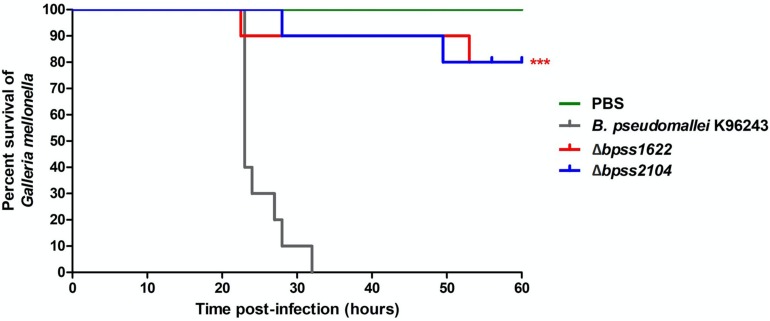
Virulence of *Burkholderia pseudomallei* strains in *Galleria mellonella* larvae. Groups of 10 larvae were challenged with the strains indicated. The experiment was performed in triplicate and showed similar results. A representative result is shown as the percentage of surviving larvae from 24 to 60 h after infection with *B. pseudomallei* K96243 wild type (gray), Δ*bpss1622* mutant, or Δ*bpss2104* mutant. *P*-values were determined by the log rank (Mantel–Cox) test, and triple asterisks denote a significant difference of *P* < 0.0001 between the wild type and mutants.

We selected two genes in Group B that had not previously been mutated, *bpss1622* (an inner rod component of Type III secretion system cluster 2) and *bpss2104* (a component of basal secretion apparatus of Type IV secretion system cluster 3) as targets for deletion in *B. pseudomallei* K96243 because these genes have not previously been investigated for a role in virulence. Double-recombinants lacking the targeted regions were validated by PCR. The wild-type strain and the mutants were then tested for virulence in *G. mellonella* larvae. No deaths were observed when larvae were injected with PBS (Figure 5). At 32 h post-challenge, wild-type *B. pseudomallei* K96243 caused 100% mortality but the *B. pseudomallei*Δ*bpss1622* or Δ*bpss2104* mutants caused only 10% mortality (Figure 5). While this implicates the targeted macrophage-induced regions in virulence, both genes are part of operons and further research will be required to determine if observed phenotypes are attributable to the genes *per se*, or polar effects.

## Discussion

We have established a promoter trap library and used DFI to identify *B. pseudomallei* genes induced during macrophage infection. Previously, *B. pseudomallei* genes which are differentially expressed in broth and in human macrophage-like cells as measured using a DNA microarrays, has been reported (Chieng et al., 2012). Both microarray and DFI technologies detect differential gene expression at the level of transcription. Microarrays measure the levels of mRNA directly whereas the approach we have used measures promoter activity using a fluorescent reporter. Each approach has advantages and limitations. A limitation of microarrays is the difficulty of detecting low level gene expression. Additionally, because mRNA is often unstable, genes which show temporal upregulation may not be identified using methods which directly measure mRNA levels, unless samples are taken frequently. In contrast, the use of a stable fluorescent reporter allows promoter activity to be measured retrospectively. Finally, reporter-based methods are of value where there is a need to study gene expression in individual infected cells (Valdivia and Falkow, 1997).

Our library showed 88% genome coverage of the *B. pseudomallei* K96243 genome, but, because the DNA fragments were generated after partial cleavage with restriction endonuclease, some promoters may not have been cloned or obtained in the correct orientation relative to the reporter gene. As a result, some macrophage-induced promoters were likely not detected in this study. Also, our strategy for screening for promoter activity in *B. thailandensis* assumes that gene regulation is similar in *B. thailandensis* and *B. pseudomallei* and this may not be the case for all genes. For example, the arabinose assimilation operon is differentially represented in these two species, with consequences for the expression of the Bsa Type III secretion system (Moore et al., 2004).

To identify promoters induced in host cells, we screened *B. thailandensis*-infected macrophages at a time point in the infection cycle at which phagosome escape and actin-based motility have been reported (Stevens J.M. et al., 2005; Wand et al., 2011), but prior to cell fusion resulting in the formation of multinucleate giant cells (MNGCs). To avoid the possibility that fluorescent macrophages were infected by multiple bacterial clones we used three cycles of infection and cell sorting. After that, the recovered *B. thailandensis* eGFP-positive clones were propagated by culturing in LB broth for plasmid preparation. This *in vitro* growth stage might affect the abundance of some clones leading to the enrichment of false negatives but not false positives. Our microarray data, which were derived from the library grown *in vitro*, showed comprehensive coverage of the genome. Any selection imposed by growth in LB broth was therefore not obvious from our analysis of library diversity.

After sequencing the clones recovered from fluorescent macrophages, the short reads obtained were aligned with the *B. pseudomallei* K96243 genome. This revealed whether the 5′ end of the fragment was upstream or within the predicted open reading frame (ORF) of a gene. Typically, a promoter would be located upstream of the ORF. In this study, most of the promoters were mapped within an annotated gene. However, it is possible that the gene annotation is incorrect or that an internal promoter is present. Internal promoters have been reported in a range of bacteria (Miyata et al., 2013; Namprachan-Frantz et al., 2014; Perault and Cotter, 2018). For example, the *Streptococcus pyogenes* salivaricin (*sal*) operon is regulated by a promoter upstream of the operon and also by a second promoter located within the operon (Namprachan-Frantz et al., 2014). Internal promoters are also found in genes encoding immunity proteins of *Vibrio cholerae*, which are independently expressed of other proteins encoded within the same operon (Miyata et al., 2013). Another example found in *Burkholderia dolosa* where a gene internal promoter is involved in expression of the *Burkholderia*-type contact-dependent growth inhibition (CDI) system-encoding locus (*bcpAIOB*). The region upstream of the *B. dolosa bcp-3* operon does not show promoter activity and fails to drive the expression of the downstream genes. However, promoter activity was detected when the 500 bp distal region of the first gene in the *bcp-3* operon was cloned into a *lacZ* expression vector, indicating that a promoter resides at the 3′ end of the first gene of the operon (Perault and Cotter, 2018).

In this study, we identified 138 genomic regions inferred to possess promoter activity during J774A.1 macrophage infection. A previous microarray study carried out at 1, 2, 4, or 6 h after infection of U937 macrophages revealed 25 *B. pseudomallei* genes that were upregulated (Chieng et al., 2012). Only *bpss0143*, encoding a transcriptional regulator, was identified in both studies. However, our qRT-PCR analysis also showed differences compared to the previously reported microarray based study (Chieng et al., 2012). For example, the increased expression of *bpss1835*, encoding an LPS biosynthesis mannose-1-phosphate guanylyl transferase, was confirmed by qRT-PCR in our study but this gene showed reduced expression at 6 h post infection in human U937 cells, compared to *in vitro* culture (Chieng et al., 2012). This could reflect differences in the intracellular environment in U937 human macrophages and in J774A.1 mouse macrophages. Or it may indicate differences in gene regulation in *B. thailandensis* and *B. pseudomallei*. Alternatively, the fundamental differences in microarray and qRT-PCR methodologies could be responsible for these differences.

A greater proportion of the macrophage-induced promoters identified in this study were located on chromosome 2 of *B. pseudomallei* K96243. This chromosome encodes many accessory functions, including functions associated with adaptation and survival in different niches and well-characterized virulence genes (Holden et al., 2004). Thirteen genes downstream of the promoters we identified are proximal to genes which have previously been shown to be involved in oxidative stress responses (Jitprasutwit et al., 2014). These include genes upregulated when *B. pseudomallei* K96243 is exposed to hydrogen peroxide (H_2_O_2_) and which might play a role in survival in macrophages (Jitprasutwit et al., 2014). It was also noticeable that a fifth of the genes we identified in this study (28 genes) have previously been reported in a transposon-directed insertion site sequencing (TraDIS) library as genes essential for the *in vitro* growth of *B. pseudomallei* K96243 (Moule et al., 2014). Although *in vitro* induced *B. pseudomallei* promoters should be excluded in our study, we found that *bpss0547*, which was from this *in vitro* TraDIS study, showed increased expression after macrophage infection in our study. Therefore, our findings suggest that some essential genes may also play a role in virulence. Approximately one fifth of the macrophage-induced promoters we identified (27 promoters) have been reported to co-locate with virulence-related genes shared by *B. pseudomallei* and *Burkholderia mallei* (Schell et al., 2008) although four of the genes (*bpsl1057*, *bpss0796*, *bpss0822*, and *bpss1268*) are absent in *B. thailandensis* (Schell et al., 2008). The roles of these genes have already been reported. For example, *bpss0796* encoding a putative trimeric autotransporter protein (BoaA) plays a role in survival of *B. pseudomallei* in macrophages (Balder et al., 2010), and a *boaA* mutant was attenuated in BALB/c mice (Lazar Adler et al., 2015). In addition, BPSS0796 is also identified as an immunogenic protein in melioidosis patient serum (Tiyawisutsri et al., 2007). Collectively, these data support our proposal that the genes co-located with the promoters we have identified play a role in the virulence of *B. pseudomallei*. Our proposal is also supported by the finding that *bpss1622* or *bpss2104* deletion mutants were attenuated in wax moth larvae.

Our promoter trap library and DFI screening strategy enabled us to identify *B. pseudomallei* promoters induced during infection of mouse macrophages. A number of novel *B. pseudomallei* genes induced during macrophage infection that have not been reported in the previous literature were found. In comparison with antibiotic resistance-based IVET that requires an appropriate concentration to isolate promoters that are active at a specific level, promoters with transient or weak activity can be detected using DFI (Rediers et al., 2005). A promoter trap screen using fusions to a promoterless chloramphenicol resistance gene integrated into the *B. pseudomallei* genome identified 15 different genomic loci after four rounds of RAW264.7 macrophage infection using an MOI of 100 (Shalom et al., 2007). Among these were three genes (*tssH-5*, *tssI-5*, and *tssM-5*) located within the same Type VI protein secretion system cluster (*tss-5*), *mntH*, encoding a natural resistance-associated macrophage protein (NRAMP)-like manganese ion transporter, and *bhuT*, a heme acquisition gene (Shalom et al., 2007). No overlap was detected in the genes identified in the present study. Our dataset complements other datasets of potential virulence-associated genes in *B. pseudomallei* and opens new opportunities to investigate the roles of the genes we have identified in intracellular life and disease.

## Data Availability Statement

The datasets GENERATED for this study can be found in the SNBI SRA accession PRJNA599542.

## Author Contributions

SJ, RT, and SK contributed to the conception and design of the study. SJ, CH, NO, PW, VM, PV, JS, and CO performed the experiments. SJ and NJ analyzed the data and wrote the manuscript. PV, JS, and MS provided technical guidance and suggested and commented on the design of the experiments. RT, MS, and SK edited the manuscript. All authors read and approved the final version of the manuscript.

## Conflict of Interest

The authors declare that the research was conducted in the absence of any commercial or financial relationships that could be construed as a potential conflict of interest.
