# Supplementary material for: Identification of Burkholderia pseudomallei Genes Induced During Infection of Macrophages by Differential Fluorescence Induction
Source: Front Microbiol. 2020 Feb 21;11:72. doi: 10.3389/fmicb.2020.00072 (PMC7047822; doi:10.3389/fmicb.2020.00072)
Supplement: Supplementary file 1 [file Image_1.pdf]

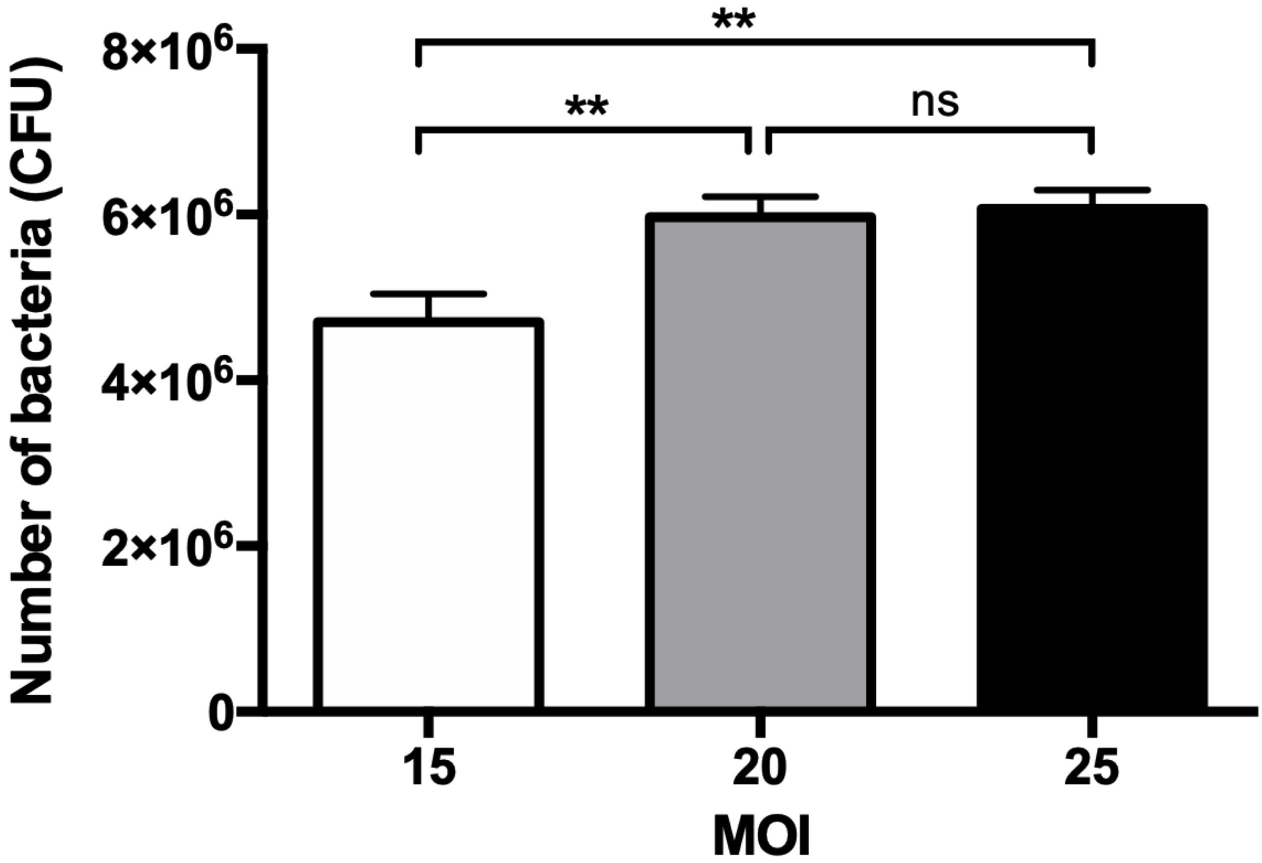

Supplementary Figure 1. Titration of MOI for macrophage infection with *B. thailandensis*. After infection of J774A.1 cells at different MOIs, the numbers of intracellular *B. thailandensis* were quantified. Values shown are the mean of three independent experiments. Asterisks indicate significant differences ($P < 0.05$).
