# Supplementary material for: Identification of Burkholderia pseudomallei Genes Induced During Infection of Macrophages by Differential Fluorescence Induction
Source: Front Microbiol. 2020 Feb 21;11:72. doi: 10.3389/fmicb.2020.00072 (PMC7047822; doi:10.3389/fmicb.2020.00072)
Supplement: Supplementary file 2 [file Image_2.pdf]

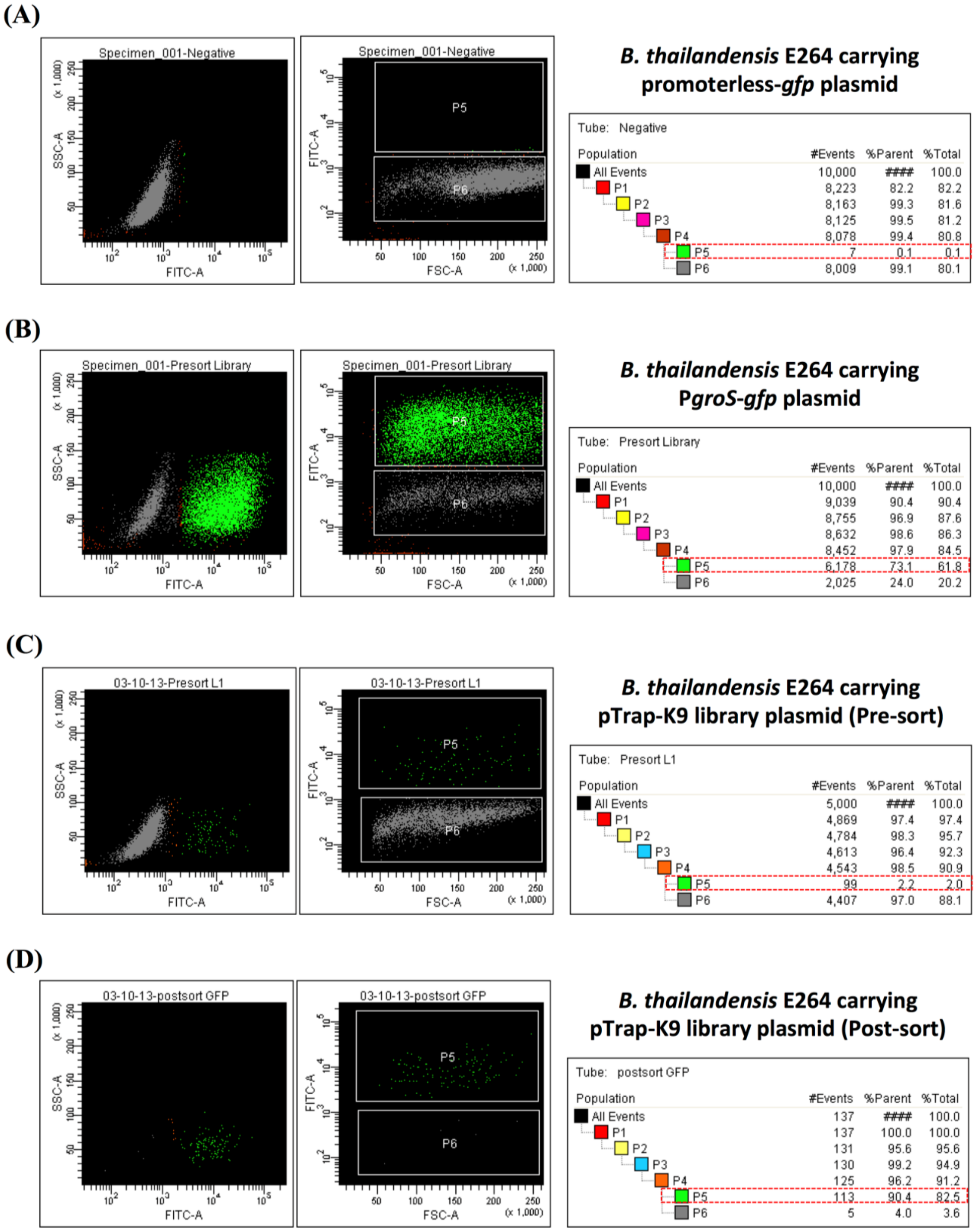

Tube: Negative

Population	#Events	%Parent	%Total
All Events	10,000	####	100.0
P1	8,223	82.2	82.2
P2	8,163	99.3	81.6
P3	8,125	99.5	81.2
P4	8,078	99.4	80.8
P5	7	0.1	0.1
P6	8,009	99.1	80.1

Tube: Presort Library

Population	#Events	%Parent	%Total
All Events	10,000	####	100.0
P1	9,039	90.4	90.4
P2	8,755	96.9	87.6
P3	8,632	98.6	86.3
P4	8,452	97.9	84.5
P5	6,178	73.1	61.8
P6	2,025	24.0	20.2

Tube: Presort L1

Population	#Events	%Parent	%Total
All Events	5,000	####	100.0
P1	4,869	97.4	97.4
P2	4,784	98.3	95.7
P3	4,613	96.4	92.3
P4	4,543	98.5	90.9
P5	99	2.2	2.0
P6	4,407	97.0	88.1

Tube: postsort GFP

Population	#Events	%Parent	%Total
All Events	137	####	100.0
P1	137	100.0	100.0
P2	131	95.6	95.6
P3	130	99.2	94.9
P4	125	96.2	91.2
P5	113	90.4	82.5
P6	5	4.0	3.6

Supplementary Figure 2. Flow cytometric analysis of eGFP expression in J774A.1 macrophages infected with *B. thailandensis* E264 carrying promoterless-gfp plasmid as a control for eGFP-negative cells **(A)**, a constitutive eGFP expression plasmid (pBHR4-*groS*-eGFP) as a control for eGFP-positive cells **(B)**. Macrophages infected with the *B. thailandensis* carrying pTrap-K9 library before cell sorting **(C)** and after cell sorting **(D)**.
