# Supplementary material for: Identification of Burkholderia pseudomallei Genes Induced During Infection of Macrophages by Differential Fluorescence Induction
Source: Front Microbiol. 2020 Feb 21;11:72. doi: 10.3389/fmicb.2020.00072 (PMC7047822; doi:10.3389/fmicb.2020.00072)
Supplement: Supplementary file 3 [file Table_1.pdf]

Supplementary Table 1. Primers used in this study

Primers	Nucleotide sequences (5' to 3')	Purpose
**Derived from the pBHR4 plasmid sequence**		
Trap-F	AAGGAGCTCAGATCTACTAGTATGGTGTCG AAGGG	Amplification of eGFP gene and construction of promoterless-*gfp* plasmid
Trap-R	TCTAGAGGATCCCCGGGTAC	Amplification of eGFP gene and construction of promoterless-*gfp* plasmid
**Derived from the *B. pseudomallei* genome**		
23S-F	TTTCCCGCTTAG ATG CTTT	qPCR Normalization
23S-R	AAAGGTACTCTGGGGATAA	qPCR Normalization
cydB-F	GATCCGAAGAGCAGCC	qPCR Normalization
cydB-R	CAGCCCGTGTAGAGCAG	qPCR Normalization
bpsl0007-F	AACGCGTTCAATCAGGG	qPCR
bpsl0007-R	TCAGATTGACGATGCTGTTG	qPCR
bpsl0125-F	CTTTGGAACTACGAGCCG	qPCR
bpsl0125-R	GAGCTCGAGAATGTGGC	qPCR
bpsl0346-F	GCTGCGAAATTCTGATTGT	qPCR
bpsl0346-R	CGAACAGGAAGCGATTTTC	qPCR
bpsl0479-F	GAACGACGACAACAACCTGC	qPCR
bpsl0479-R	ACGTGCCTTCTTCGAGATACC	qPCR
bpsl1534-F	CGTGCATCAACAAGTTCTAC	qPCR
bpsl1534-R	TTCGGACGAAGAAACGAAAA	qPCR
bpsl2987-F	CAAGGTGCTGAACATCGT	qPCR
bpsl2987-R	TACGTCACCTTGTCGTTTTC	qPCR
bpsl3338-F	GGCTCAGATCGTCGGAGAGA	qPCR
bpsl3338-R	GGCGGGTTCACCAATGTCTA	qPCR
bpss0547-F	CCCTTCTTCTCGCAATCGCT	qPCR
bpss0547-R	GTACTGGAGCGCGTTGAACC	qPCR
bpss0769-F	AGCACCGGATAGAGCAG	qPCR
bpss0769-R	CACATCGAGCTCGGTAAC	qPCR
bpss1039-F	ATTGCTGATCAGGTTGCTG	qPCR
bpss1039-R	CCAGTTCTCGTCGAATCTC	qPCR
bpss1268-F	CCTGATACTCGCCCTGCC	qPCR
bpss1268-R	CTGGCTCAAGCACATGAAGG	qPCR
bpss1442-F	CTGGCTCAAGCACATGAAGG	qPCR
bpss1442-R	CATCGGGTAATGGCAGGTCT	qPCR
bpss1498-F	TCAAGGTCAAAGGAAAAAC	qPCR
bpss1498-R	AAGGCGAGGATGTGGAT	qPCR
bpss1835-F	AGGACGTGAAGAAAATCGTC	qPCR
bpss1835-R	TGTCGTCCATAGTTGTCTTC	qPCR
**For deletion mutagenesis study**		

Primers	Nucleotide sequences (5’ to 3’)	Purpose
US1622-F	ATATATACTAGTACGAAGTCGAGCGCGGTG	Amplify 400 bp upstream gene of *bpss1622* and merodiploid selection
US1622-R	ATATATGGGCCCGGTGTGCGCTCCGTGTC	
DS1622-F	ATATATGGGCCCCGAGGAGCTCCGCATGAACA	Amplify 400 bp downstream gene of *bpss1622* and merodiploid selection
DS1622-R	ATATATTCTAGAGTCCTTCGTCGACGTGACGA	
BPSS1622-F0	ATGGCCGAAGAGAAAACCGAAGA	Δ*bpss1622* mutant selection
BPSS1622-R0	TCACTGGTTCTTCATCAGCGAGC	
US2104-F	ATATATACTAGTACCGCAGCGTGATCGTG	Amplify 400 bp upstream gene of *bpss2104* and merodiploid selection
US2104-R	ATATATTCTAGAGCTCAGGGCGCCTCCTTC	
DS2104-F	ATATATTCTAGAGCACGATGCAGACGACGC	Amplify 400 bp downstream gene of *bpss2104* and merodiploid selection
DS2104-R	ATATATGCATGCCGGATCGCATCGAGCAGC	
BPSS2104-F0	CCGCGCGTCGCGAAGTT	Δ*bpss2104* mutant selection
BPSS2104-R0	GCACGAGCCGCACCTTCG	
