# Supplementary material for: Identification of Burkholderia pseudomallei Genes Induced During Infection of Macrophages by Differential Fluorescence Induction
Source: Front Microbiol. 2020 Feb 21;11:72. doi: 10.3389/fmicb.2020.00072 (PMC7047822; doi:10.3389/fmicb.2020.00072)
Supplement: Supplementary file 4 [file Table_2.pdf]

Supplementary Table 2. *B. pseudomallei* genes located downstream of putative macrophage-induced promoters found in this study.

Group A: The start of the mapped regions of the identified genes in this group was upstream of the translational start site.

No.	Gene ID	Strand	Product	Position / total gene in operon[a]	Number of reads	Length of mapped region (bp)	Reads mapped within CDS (bp)	Gene length (bp)	% coverage	Distance[b]
1.	BPSL0007 (gspD)*	+	General secretory pathway protein D	1/3	31	182	122	2274	5.37%	-60
2.	BPSL0125 (sun)*	+	NOL1/NOP2/Sun family protein	1/4	31	229	222	1410	15.74%	-7
3.	BPSL0266	-	Hypothetical protein	1/1	268	156	33	222	14.86%	-123
4.	BPSL0346*	-	Dihydrodipicolinate synthetase family protein	1/1	76	96	42	903	4.65%	-54
5.	BPSL0922	+	Putative ABC transporter ATP-binding protein	3/4	31	198	87	774	11.24%	-111
6.	BPSL1057	+	Hypothetical protein	1/1	26	54	7	477	1.47%	-47
7.	BPSL1534 (phbC)*	+	Poly-beta-hydroxybutyrate polymerase	1/2	27	293	276	1806	15.28%	-17
8.	BPSL1603	-	Putative lipoprotein	4/8	15	159	117	531	22.03%	-42
9.	BPSL2865 (katG)	-	Catalase-peroxidase protein	1/1	3	177	82	2247	3.65%	-95
10.	BPSL2987 (tpx)*	+	Thiol peroxidase	6/6	121	342	257	504	50.99%	-85
11.	BPSL3010 (gspO)	+	Type IV prepilin leader peptide type M1	3/4	143	180	123	930	13.23%	-57
12.	BPSS0007	-	Hypothetical protein	1/1	43	116	43	654	6.57%	-73
13.	BPSS0343	-	Aminotransferase protein	1/1	551	171	148	1110	13.33%	-24
14.	BPSS0479*	+	Ribonucleotide reductase protein	1/1	14	195	51	2535	2.01%	-144
15.	BPSS0504	-	Hypothetical protein	1/1	27	131	67	1074	6.24%	-64

No.	Gene ID	Strand	Product	Position / total gene in operon[a]	Number of reads	Length of mapped region (bp)	Reads mapped within CDS (bp)	Gene length (bp)	% coverage	Distance[b]
16.	BPSS0646	-	Hypothetical protein	2/2	6	137	21	327	6.42%	-116
17.	BPSS0769*	-	Hypothetical protein	1/2	273	175	60	981	6.12%	-115
18.	BPSS0822	+	Hypothetical protein	4/6	47	136	1	903	0.11%	-135
19.	BPSS0951	-	ABC transporter system, ATP-binding protein	4/5	461	70	49	870	5.63%	-22
20.	BPSS1442*	+	Hypothetical protein	2/3	1362	257	109	1158	9.41%	-150
21.	BPSS1740 (lipB)	-	Lipase chaperone	2/2	2	59	1	1035	0.10%	-60
22.	BPSS1835*	+	LPS biosynthesis mannose-1-phosphate guanylyltransferase	1/1	4	224	171	1461	11.70%	-53
23.	BPSS1836	-	Hypothetical protein	1/1	16	62	56	1005	5.57%	-6
24.	BPSS1842	-	Hypothetical protein	2/3	3463	125	13	540	2.41%	-113
25.	BPSS2039	+	Cyclopropane-fatty-acyl-phospholipid synthase	3/6	46	250	189	936	20.19%	-61
26.	BPSS2192	+	Hypothetical protein	3/9	47	166	39	1191	3.27%	-127
27.	BPSS2326	-	Flavin-binding monooxygenase-like protein	3/10	26	390	340	1488	22.85%	-50

Group B: The start of the mapped regions of the identified genes in this group was resided within the CDS.

No.	Gene ID	Strand	Product	Position / total gene in operon[a]	Number of reads	Read length (bp)	Reads mapped on the gene (bp)	Gene length (bp)	% coverage	Distance[b]
**DISTANCE IS LESS THAN 100 BP**										
1.	BPSL1075	+	Putative transport system, integral membrane protein	1/2	3236	178	284	1314	21.61%	68
2.	BPSL1130	-	Putative sigma factor	1/1	94	142	142	894	15.88%	68

No.	Gene ID	Strand	Product	Position / total gene in operon[a]	Number of reads	Read length (bp)	Reads mapped on the gene (bp)	Gene length (bp)	% coverage	Distance[b]
3.	BPSL1189	-	Putative kinase	7/7	219	102	102	471	21.66%	63
4.	BPSL1809	-	Putative amino acid transport system, exported protein	1/3	490	240	242	816	29.66%	72
5.	BPSL1904	+	Hypothetical protein	1/1	871	96	174	483	36.02%	46
6.	BPSL2269 (ftsB)	-	Cell division protein	2/2	3	125	125	432	28.94%	35
7.	BPSL2427 (recO)	-	DNA repair protein	3/6	4	67	67	861	7.78%	92
8.	BPSL2473 (thyA)	-	Putative thymidylate synthase	2/2	129	67	67	972	6.89%	15
9.	BPSL2928 (purB)	+	Adenylosuccinate lyase	2/2	618	157	160	1452	11.02%	59
10.	BPSL3319 (fliC)	+	Flagellin	1/1	1229	67	156	1167	13.37%	80
11.	BPSL3338*	-	Putative methyl-accepting chemotaxis protein	1/1	52	191	191	1818	10.51%	20
12.	BPSS0023	+	Cytochrome monooxygenase related protein	1/1	1026	110	111	1407	7.89%	55
13.	BPSS0228	-	Hypothetical protein	1/1	1206	84	87	264	32.95%	15
14.	BPSS0293	+	Multidrug-efflux transporter protein	3/4	2445	61	63	3186	1.98%	77
15.	BPSS0511	-	Hypothetical protein	3/7	20	154	154	1002	15.37%	17
16.	BPSS1039 (irlS)*	-	Transmembrane invasion-related two-component sensor protein	5/5	9	223	223	1395	15.99%	2
17.	BPSS1156 (narI)	-	Respiratory nitrate reductase subunit	1/3	179	108	110	684	16.08%	87
18.	BPSS1268*	-	Efflux system protein	3/5	1666	90	90	1290	6.98%	3
19.	BPSS1622**	+	Type III secretion protein	1/8	3699	59	66	582	11.34%	56
20.	BPSS1715 (gltA)	-	Citrate synthase	8/8	326	118	119	1302	9.14%	65
21.	BPSS1820	-	Hypothetical protein	2/2	8452	109	111	702	15.81%	88
**DISTANCE IS MORE THAN 100 BP**										
22.	BPSL0074 (dnaN)	-	DNA polymerase III subunit beta	2/3	916	60	60	1104	5.43%	103
23.	BPSL0542	+	NUDIX domain family protein	4/5	1127	131	67	858	7.81%	791
24.	BPSL0634	+	Putative oxidoreductase	1/5	1074	170	257	4080	6.30%	2183

No.	Gene ID	Strand	Product	Position / total gene in operon[a]	Number of reads	Read length (bp)	Reads mapped on the gene (bp)	Gene length (bp)	% coverage	Distance[b]
25.	BPSL0676	+	Putative transmembrane transporter protein	1/1	106	161	161	1173	13.73%	227
26.	BPSL0687 (glpK)	-	Glycerol kinase	2/3	442	130	164	1503	10.91%	1144
27.	BPSL1028 (tnpA)	-	Transposase	1/1	232	77	77	1221	6.31%	118
28.	BPSL1134	+	Hypothetical protein	1/2	682	72	19	681	2.79%	662
29.	BPSL1571	-	Hypothetical protein	1/1	22	352	345	1071	32.21%	620
30.	BPSL1647	+	Putative betaine aldehyde dehydrogenase	2/11	39	58	58	1473	3.94%	812
31.	BPSL1661	+	Putative hemolysin-related protein	3/7	34	133	133	9690	1.37%	5537
32.	BPSL1824	-	Putative ABC transport system, substrate-binding protein	1/2	966	58	59	1047	5.64%	873
33.	BPSL1932 (tnpA)	+	Transposase	1/1	272	77	77	1221	6.31%	118
34.	BPSL2243 (cysS)	-	Cysteinyl-trna synthetase	1/2	73	51	26	1398	1.86%	1372
35.	BPSL2274	-	Hypothetical protein	4/5	12	61	61	2571	2.37%	1112
36.	BPSL2488 (tnpA)	-	Transposase	1/1	8	77	77	1221	6.31%	118
37.	BPSL2502	-	ABC transporter, membrane permease	2/3	395	123	55	654	8.41%	599
38.	BPSL2511 (udg)	-	Putative UDP-glucose dehydrogenase	1/4	71	160	160	1401	11.42%	617
39.	BPSL2512	-	Hypothetical protein	2/2	156	35	36	1170	3.08%	164
40.	BPSL2552	-	Putative lipoprotein	2/3	2297	64	67	1272	5.27%	789
41.	BPSL2659 (ureC)	+	Urease alpha subunit	3/6	1911	121	122	1707	7.15%	803
42.	BPSL2817 (tnpA)	-	Transposase	1/1	8	77	77	1221	6.31%	118
43.	BPSL2843 (glcD)	+	Putative glycolate oxidase subunit glcd	2/2	25	46	48	1494	3.21%	284

No.	Gene ID	Strand	Product	Position / total gene in operon[a]	Number of reads	Read length (bp)	Reads mapped on the gene (bp)	Gene length (bp)	% coverage	Distance[b]
44.	BPSL2897 (ruvC)	+	Holliday junction resolvase	1/4	67	183	183	543	33.70%	259
45.	BPSL2925	-	Putative glutamate dehydrogenase	1/1	360	81	85	1305	6.51%	508
46.	BPSL2930	-	Putative gluconate permease	1/2	18	109	109	1362	8.00%	1139
47.	BPSL3101 (clpB)	-	Protease associated atpase clpb	4/5	81	215	216	2670	8.09%	691
48.	BPSL3144	-	Putative ABC transporter ATP-binding subunit	1/19	226	51	51	942	5.41%	876
49.	BPSL3168 (aroB)	-	3-dehydroquinate synthase	3/9	129	33	34	1080	3.15%	522
50.	BPSL3180	-	Putative cytochrome C biogenesis protein	1/2	6651	40	41	2100	1.95%	476
51.	BPSL3323	+	Putative transferase	2/8	3665	58	61	1149	5.31%	266
52.	BPSL3342	-	Putative bacteriophage protein	1/1	64	118	118	1563	7.55%	1038
53.	BPSL3344	-	Putative bacteriophage integrase	1/2	32	140	140	855	16.37%	550
54.	BPSL3349	-	Hypothetical protein	1/4	103	329	435	1734	25.09%	195
55.	BPSL3423	-	Putative Asnc-family transcriptional regulator	1/1	197	72	73	552	13.22%	321
56.	BPSS0092	+	Fimbria-related chaperone	2/2	71	518	441	738	59.76%	297
57.	BPSS0126	+	Transport system, membrane protein	1/2	354	113	113	1326	8.52%	716
58.	BPSS0130	+	Peptide synthase protein	1/3	1136	211	212	2808	7.55%	1973
59.	BPSS0141	-	Sugar ABC transport system, membrane protein	6/7	695	45	46	1035	4.44%	977
60.	BPSS0143	-	ROK family transcriptional regulator	4/7	53	78	78	1251	6.24%	815
61.	BPSS0159	+	Hypothetical protein	1/1	79	62	65	2898	2.24%	803
62.	BPSS0238	+	Penicillin-binding protein	1/2	435	133	134	2163	6.20%	833
63.	BPSS0241	-	Hemin ABC transport system, membrane protein	1/1	89	97	98	1122	8.73%	602
64.	BPSS0243	-	Hemin ABC transport system-related protein	2/3	85	49	49	1152	4.25%	806
65.	BPSS0288	+	Lipoprotein	1/1	63	173	173	1062	16.29%	131
66.	BPSS0300	-	Malonyl coa-acyl carrier protein	10/11	10	68	68	900	7.56%	734

No.	Gene ID	Strand	Product	Position / total gene in operon[a]	Number of reads	Read length (bp)	Reads mapped on the gene (bp)	Gene length (bp)	% coverage	Distance[b]
67.	BPSS0303	-	Diaminopimelate decarboxylase	7/11	64	97	97	1260	7.70%	371
68.	BPSS0311	-	Multifunctional polyketide-peptide syntase	1/2	489	43	44	8529	0.52%	3662
69.	BPSS0323	+	Hypothetical protein	3/3	13	157	100	726	13.77%	626
70.	BPSS0339	-	Amino acid dioxygenase	1/1	26	315	315	2055	15.33%	1183
71.	BPSS0424	+	Glycosyl transferase	2/2	27	86	58	1062	5.46%	1004
72.	BPSS0484 (fabH)	+	3-oxoacyl-(acyl carrier protein) synthase	4/5	23	219	219	999	21.92%	576
73.	BPSS0523	+	Hypothetical protein	6/6	20	67	67	2292	2.92%	441
74.	BPSS0524	+	Hypothetical protein	1/10	73	87	87	2289	3.80%	441
75.	BPSS0547 (glyA)*	+	Serine hydroxyl-methyltransferase	1/2	121	94	94	1290	7.29%	987
76.	BPSS0579 (braG)	+	High-affinity branched-chain amino acid transport ATP-binding protein	5/5	193	92	93	702	13.25%	235
77.	BPSS0694 (hpcC)	+	5-carboxymethyl-2-hydroxymuconate semialdehyde dehydrogenase	3/7	6	210	210	1464	14.34%	921
78.	BPSS0705	+	Response regulator protein	1/2	156	170	170	672	25.30%	108
79.	BPSS0740	-	Hypothetical protein	1/1	210	156	156	786	19.85%	436
80.	BPSS0796	+	H-NS-like protein	1/1	20	95	95	4962	1.91%	929
81.	BPSS0913	+	Methionine gamma-lyase	1/1	8	125	125	1302	9.60%	848
82.	BPSS0960	+	Rhs-related membrane protein	3/4	797	169	233	4626	5.04%	3351
83.	BPSS0965	+	Oxalate decarboxylase	1/1	952	91	93	1263	7.36%	620
84.	BPSS1014	-	Hypothetical protein	1/1	47	157	158	1194	13.23%	690
85.	BPSS1038	+	Hypothetical protein	1/1	95	123	123	273	45.05%	113
86.	BPSS1048	+	Hypothetical bacteriophage protein	2/2	161	95	97	783	12.39%	137
87.	BPSS1082	+	Bacteriophage protein gp17	5/9	98	89	90	2364	3.81%	838
88.	BPSS1333	+	Hypothetical protein	1/1	244	60	60	861	6.97%	106
89.	BPSS1350	-	Efflux/sugar transport/multidrug resistance protein	2/2	121	190	190	1587	11.97%	245
90.	BPSS1386	-	ATP/GTP binding protein	1/1	480	37	40	3834	1.04%	111

No.	Gene ID	Strand	Product	Position / total gene in operon[a]	Number of reads	Read length (bp)	Reads mapped on the gene (bp)	Gene length (bp)	% coverage	Distance[b]
91.	BPSS1405 (sctS)	+	Type III secretion-associated protein	4/4	167	37	39	264	14.77%	182
92.	BPSS1452	+	Copper nitrite reductase protein	1/1	2211	92	98	1464	6.69%	908
93.	BPSS1526 (bapC)	-	Invasion protein	3/3	75	116	116	564	20.57%	170
94.	BPSS1566	+	Probable phosphate transporter	1/1	64	567	567	1587	35.73%	503
95.	BPSS1717 (sdhB)	-	Succinate dehydrogenase catalytic subunit	6/8	104	64	64	702	9.12%	608
96.	BPSS1770 (polA)	-	DNA polymerase I	1/1	77	349	350	2772	12.63%	1088
97.	BPSS1886	-	Aromatic hydrocarbons catabolism-related dioxygenase	3/4	341	91	91	330	27.58%	224
98.	BPSS1898 (fadH)	-	Ubiquinol oxidase polypeptide I	1/2	70	82	82	2034	4.03%	659
99.	BPSS1907	-	Hypothetical protein	1/1	246	111	111	909	12.21%	546
100.	BPSS1912 (tnpA)	-	IS1001 transposase	1/1	8	77	77	1221	6.31%	118
101.	BPSS1915	+	Metallo-beta-lactamase family protein	1/1	238	196	8	1380	0.58%	1378
102.	BPSS1956	+	Acetate kinase	3/3	15	137	137	1179	11.62%	338
103.	BPSS1995 (irlS2)	+	Metal-related two-component system, histidine kinase	3/4	3098	52	198	1455	13.61%	506
104.	BPSS2015	-	Inner membrane glycosyltransferase	1/4	14	267	267	1563	17.08%	578
105.	BPSS2102	-	Protein kinase	1/12	124	89	90	2616	3.44%	1391
106.	BPSS2104**	-	Hypothetical protein	4/5	537	151	153	3630	4.21%	1532
107.	BPSS2200 (tyrB2)	+	Aspartate aminotransferase	1/1	319	48	50	1218	4.11%	518
108.	BPSS2218 (rpoN)	-	DNA-directed RNA polymerase subunit N	1/4	33	42	42	1476	2.85%	869
109.	BPSS2259	-	Fusion protein, ATP-binding transmembrane ABC transporter and regulatory protein	2/3	82	104	104	2967	3.51%	1750
110.	BPSS2287	+	Hypothetical protein	1/1	1632	58	241	654	36.85%	557
111.	BPSS2318	-	Hypothetical protein	1/2	593	156	157	2442	6.43%	2137

Note: *Genes are selected for validation using qRT-PCR., **Genes are selected for mutagenesis.
[a]Ooi WF, Ong C, Nandi T, Kreisberg JF, Chua HH, Sun G, et al. (2013) The Condition-Dependent Transcriptional Landscape of *Burkholderia pseudomallei*. PLoS Genet 9(9): e1003795.
[b]Distance from 5'-end of the read to the annotated start codon; a negative value indicates that the 5’-end of the mapped region was upstream of the translational start site, hence might include a transcriptional start site and a positive value indicates the 5’-end of the reads were inside of that genes.
