# Supplementary material for: Identification of Burkholderia pseudomallei Genes Induced During Infection of Macrophages by Differential Fluorescence Induction
Source: Front Microbiol. 2020 Feb 21;11:72. doi: 10.3389/fmicb.2020.00072 (PMC7047822; doi:10.3389/fmicb.2020.00072)
Supplement: Supplementary file 5 [file Table_3.pdf]

Supplementary Table 3. Literature of macrophage-induced genes of *B. pseudomallei* K96243 found in this study.

No.	*Sanger ID (Gene name)*	*Strand*	*Product*	*Published finding*	*Group*[#]
Oxidative stress-related/ Hypoxia-induced genes					
1	BPSL0687 (*glpK*)	-	Glycerol kinase	– Up-regulated under oxidative stress [1]	**B**
2	BPSL1057	+	Hypothetical protein	– Down-regulated in sigmaE mutant under oxidative stress [1]<br>– Known as a virulence factor of Bp K96243 [2]<br>– Computationally derived virulome shared by Bp and Bm, and absent in Bth [3]	**A**
3	BPSL1075	+	Putative transport system, integral membrane protein	– BPSL1075-76 up-regulated under oxidative stress [1]	**B**
4	BPSL1904	+	Hypothetical protein	– Up-regulated under oxidative stress [1]	**B**
5	BPSL2427 (*recO*)	-	DNA repair protein	– *BPSL2426 predicted as an essential gene for in vitro growth of Bp K96243* [4]<br>– *BPSL2426 reported as an oxidative(H_2O_2) stress responsive protein* [5]	**B**
6	BPSL2865 (*katG*)	-	Catalase-peroxidase protein	– Increased expression during exposure to H_2O_2-induced oxidative stress [1]<br>– A *katG* insertion mutant was hypersensitive to various oxidants [6]<br>– Known as a virulence factor of Bp K96243 [2]	**A**
7	BPSL2925	-	Putative glutamate dehydrogenase	– Up-regulated under oxidative stress and sigma E independent [1]<br>– Predicted as an essential gene for *in vitro* growth of Bp K96243 [4]	**B**
8	BPSL2928 (*purB*)	+	Adenylosuccinate lyase	– Up-regulated under oxidative stress [1]<br>– Predicted as an essential gene for *in vitro* growth of Bp K96243 [4]	**B**

No.	***Sanger ID (Gene name)***	***Strand***	***Product***	***Published finding***	***Group***[#]
9	BPSL2930	-	Putative gluconate permease	– Up-regulated under oxidative stress [1]<br>– *BPSL2929 known as a virulence factor of Bp K96243* [7]	**B**
10	**BPSL2987* (*tpx*)**	+	Thiol peroxidase	– *P. aeruginosa* tpx plays a role in protecting the bacteria from H_2O_2 [8]	**A**
11	BPSL3144	-	Putative ABC transporter ATP-binding subunit	– Up-regulated under oxidative stress [1]<br>– *BPSL3128 similar to tatA of Bt known as a virulence factor* [9]<br>– *BPSL3133 known as a virulence factor of Bp K96243* [10, 11]	**B**
12	**BPSL3338***	-	Putative methyl-accepting chemotaxis protein	– Identified as a hypoxia-induced genes [12]	**B**
13	BPSL3344	-	Putative bacteriophage integrase	– Up-regulated in a sigma E mutant under oxidative stress [1]	**B**
14	BPSS0238	+	Penicillin-binding protein	– Up-regulated under oxidative stress [1]	**B**
15	BPSS0323	+	Hypothetical protein	– Computationally derived virulome shared by Bp and Bm [3]<br>– Up-regulated in a sigma E mutant under oxidative stress [1]	**B**
16	BPSS0646	-	Hypothetical protein	– Up-regulated in a sigma E mutant under oxidative stress [1]	**A**
17	BPSS0913	+	Methionine gamma-lyase	– Up-regulated under oxidative stress [1]<br>– Computationally derived virulome shared by Bp and Bm [3]<br>– Recruited to Bp expressing BimA (10276 pBHR-virAG) [13]	**B**
18	BPSS0965	+	Oxalate decarboxylase	– Up-regulated in the liver, lung and spleen from i.p. infected animals [14]<br>– Down-regulated under oxidative stress but up-regulated sigma E mutant [1]	**B**
19	BPSS1156 (*narI*)	-	Respiratory nitrate reductase subunit	– Down-regulated under oxidative stress [1]	**B**
20	**BPSS1442***	+	Hypothetical protein	– Up-regulated under oxidative stress [1]<br>– *BPSS1439 identified as a cross-reactive antigen* [15]<br>– *BPSS1439 mutant demonstrated a moderate attenuation in the BALB/c murine* [16]	**A**

No.	***Sanger ID (Gene name)***	***Strand***	***Product***	***Published finding***	***Group***[#]
21	BPSS1886	-	Aromatic hydrocarbons catabolism-related dioxygenase	– Down-regulated under oxidative stress [1]<br>– *BPSS1885 predicted as an essential gene for in vitro growth of Bp K96243* [4]<br>– Down-regulated in Bp KHW, compared to *bsaN* mutant, a regulatory gene of T3SS3 and T6SS1 loci is deleted [17]	**B**
22	BPSS1896 (*cyoB*)	-	Ubiquinol oxidase polypeptide I	– BPSS1896-97 identified as a hypoxia-induced genes [12]	**B**
23	BPSS1956	+	Acetate kinase	– Identified as a hypoxia-induced gene [12]<br>– Down-regulated under oxidative stress [1]<br>– BPSS1956-54 down-regulated by a Bp transcriptional regulator under nutrient-limited conditions [18]	**B**
24	BPSS1995 (*irlS2*)	+	Metal-related two-component system, histidine kinase	– Up-regulated under oxidative stress [1]<br>– *BPSS1996 identified as a immunogenic protein react with sera from melioidosis patients* [19]	**B**
Virulence-associated genes					
25	**BPSL0125* (*sun*)**	+	NOL1/NOP2/Sun family protein	– *BPSL0127 and BPSL0128 are known as virulence factors of Bp K96243 [2, 20]*<br>– *BPSL0128 predicted as an essential gene for in vitro growth of Bp K96243 [4]*	**A**
26	BPSL0634	+	Putative oxidoreductase	– BALB/c mice infection with transposon insertion in BPSL0634 resulting in attenuation [21]<br>– *BPSL0635 down-regulated genes found in infected livers and i.n. infected liver, lungs, and spleen in hamster model* [14]	**B**
27	BPSL3168 (*aroB*)	-	3-dehydroquinate synthase	– Known as a virulence factor of Bp K96243 [7, 21]	**B**
28	**BPSS1039* (*irlS*)**	-	Transmembrane invasion-related two-component sensor protein	– Known as a virulence factor of Bp K96243 [2]<br>– BPSS1039 – BPSS1043 expressed upon zinc exposure [22]<br>– *BPSL0142 predicted as an essential gene for in vitro growth of Bp K96243* [4]	**B**
29	**BPSS1268* (*glbD*)**	-	Efflux system protein	– Known as a virulence factor of Bp K96243 [2]	**B**

No.	*Sanger ID (Gene name)*	*Strand*	*Product*	*Published finding*	*Group*#
				– Computationally derived virulome shared by Bp and Bm, and absent in Bth [3]	
In silico derived virulence-related genes					
30	BPSL1134	+	Hypothetical protein	– Computationally derived virulome shared by Bp and Bm [3]	**B**
31	BPSL1603	-	Putative lipoprotein	– Predicted as an essential gene for *in vitro* growth [4]<br>– Computationally derived virulome shared by Bp and Bm [3]<br>– *BPSL1600 identified as a cross-reactive antigen* [15]	**A**
32	BPSS0023	+	Cytochrome monooxygenase related protein	– Computationally derived virulome shared by Bp and Bm [3]	**B**
33	**BPSS0769***	-	Hypothetical protein	– Computationally derived virulome shared by Bp and Bm [3]	**A**
34	BPSS0159	+	Hypothetical protein	– Computationally derived virulome shared by Bp and Bm [3]	**B**
35	BPSS0740	-	Hypothetical protein	– Computationally derived virulome shared by Bp and Bm [3]	**B**
36	BPSS0822	+	Hypothetical protein	– Computationally derived virulome shared by Bp and Bm, and absent in Bth [3]<br>– *BPSS0823 predicted as an essential gene for in vitro growth of Bp K96243[4]*	**A**
37	BPSS0960	+	Rhs-related membrane protein	– Computationally derived virulome shared by Bp and Bm [3]	**B**
38	BPSS1452	+	Copper nitrite reductase protein	– Computationally derived virulome shared by Bp and Bm [3]	**B**
39	**BPSS1622**** **(*sctGb*)**	+	Type III secretion protein	– Computationally derived virulome shared by Bp and Bma [3]<br>– *BPSS1624 identified as a cross-reactive antigen* [15]	**B**
40	BPSS1842	-	Hypothetical protein	– Computationally derived virulome shared by Bp and Bm [3]	**A**
41	BPSS1907	-	Hypothetical protein	– Computationally derived virulome shared by Bp and Bm [3]	**B**
42	BPSS2102	-	Protein kinase	– Computationally derived virulome shared by Bp and Bm [3]<br>– *Recombinant protein BPSS2098 (Hcp3) protected 50% of mice against challenge* [23]	**B**
43	**BPSS2104****	-	Hypothetical protein	– Computationally derived virulome shared by Bp and Bm [3]	**B**
44	BPSS2318	-	Hypothetical protein	– Computationally derived virulome shared by Bp and Bm [3]	**B**

No.	Sanger ID (Gene name)	Strand	Product	Published finding	Group[#]
Sero-reactive antigens					
45	**BPSL0007* (*gspD*)**	+	General secretory pathway protein D	– Specifically expressed during human infection [19]	**A**
46	**BPSL1534* (*phbC*)**	+	Poly-beta-hydroxybutyrate polymerase	– Identified as a protein reactive against infected human sera [19]	**A**
47	BPSL1661	+	Putative hemolysin-related protein	– Predicted as an essential gene for *in vitro* growth [4]<br>– Reacted with melioidosis sera [15]	**B**
48	BPSS0143	-	ROK family transcriptional regulator	– Up-regulated during intracellular growth in host macrophages [24]<br>– *BPSS0140 identified as a cross-reactive antigen* [15]	**B**
49	BPSS0796	-	H-NS-like protein	– Identified as a cross-reactive antigen [15]<br>– Known as a virulence factor of Bp K96243 [2, 25]<br>– Identified as an immunogenic protein in melioidosis patient serum and ortholog of BPSS0796 found in Bm but not in Bt [26]<br>– Designated BoaA, an adhesin common in Bp and Bm and involved in the survival of Bp in macrophages [27]<br>– *boa* mutant increased in median lethal dose in BALB/c mice delivered by the intra-peritoneal route [25]<br>– Computationally derived virulome shared by Bp and Bm, and absent in Bth [3]	**B**
50	**BPSS1835* (*manC*)**	+	LPS biosynthesis mannose-1-phosphate guanylyltransferase	– Identified as a protein reactive against infected human sera [19]<br>– Down-regulated under oxidative stress [1]<br>– Down-regulated in liver and lungs of infected hamsters [28]<br>– Down-regulated in U937 [24]	**A**
51	BPSS1915	+	Metallo-beta-lactamase family protein	– Identified as an immunogenic protein reactive against infected human sera [19]<br>– Down-regulated under oxidative stress [1]<br>–	**B**
52	BPSS2015	-	Inner membrane glycosyltransferase	– *BPSS2013 identified as a seroreactive antigen* [15]	**B**

No.	Sanger ID (Gene name)	Strand	Product	Published finding	Group[#]
Genes expression detected in infected cell lines / tissues					
53	BPSL2269 (*ftsB*)	-	Cell division protein	– Constitutively express including in lungs of BALBc [22]<br>– *BPSL2270 predicted as an essential gene for in vitro growth [4]*	**B**
54	BPSS0141	-	Sugar ABC transport system, membrane protein	– BPSS0140 up-regulated in U937 macrophages [24]<br>– BPSS0140 identified as a cross-reactive antigen [15]	**B**
55	**BPSS0547* (*glyA*)**	+	Serine hydroxyl-methyltransferase	– Predicted as an essential gene for *in vitro* growth of Bp K96243 [4]<br>– Down-regulated genes found in infected livers and i.n. infected lungs in hamster model [14]	**B**
56	BPSS1405 (*sctS*)	+	Type III secretion-associated protein	– Up-regulated gene *in vivo* determined by DNA microarray in the spleen of BALB/c [29]	**B**
57	BPSS1526 (*bapC*)	-	Invasion protein	– Express in lungs of BALBc [22]<br>– Known as a virulence factor of Bp K96243 [2]<br>– BapC is a secreted T3SS effector that is not essential for survival within RAW 264.7 and play a minor role in the vivo growth of Bp K96243 [30]<br>– BapC mutant does not lead to attenuation in the hamster infection model [31]<br>– Up-regulated in Bp KHW, compared to *bsaN* mutant [17]<br>– Computationally derived virulome shared by Bp and Bm [3]	**B**
Motility and biofilm-regulated gene					
58	BPSL3010 (*gspO*)	+	Type IV prepilin leader peptide type M1	None	**A**
59	BPSS0092	+	Fimbria-related chaperone	– Up-regulated in a high biofilm producer strain [32]	**B**
60	BPSS0484 (*fabH*)	+	3-oxoacyl-(acyl carrier protein) synthase	– Up-regulated in a high biofilm producer strain [32]	**B**
61	BPSS1740 (*lipB*)	-	Lipase chaperone	– Up-regulated in a high biofilm producer strain [32]<br>– Down-regulated in *bprR* mutant (a response regulator gene of two-component signal transduction system mutant) [33]	**A**
Taurine-regulated genes					

No.	*Sanger ID (Gene name)*	*Strand*	*Product*	*Published finding*	*Group[#]*
62	BPSL0266	-	Hypothetical protein	– Taurine regulated genes in Bp K96243 [34]	**A**
63	BPSL3319 (*fliC*)	+	Flagellin	– Taurine regulated genes in Bp K96243 [34]<br>– Known as a virulence factor of Bp K96243 [2]<br>– Identified as a major sero-reactive antigen [15, 35]<br>– *fliC* deletion (KHWΔfliCKm) mutant was avirulent during intranasal infection of BALB/c [36]<br>– Up-regulated in sigmaE mutant [1]<br>– Down-regulated in Bp KHW, compared to *bsaN* mutant [17]	**B**
64	BPSL3323	+	Putative transferase	– Taurine regulated genes in Bp K96243 [34]<br>– BPSL3323-3329 up-regulated in *bprS* mutant [33]	**B**
65	BPSS0241	-	Hemin ABC transport system, membrane protein	– Taurine regulated genes in Bp K96243 [34]<br>– Up-regulated by curcumin [37]	**B**
66	BPSS0243	-	Hemin ABC transport system-related protein	– Taurine regulated genes in Bp K96243 [34]<br>– BPSS0243-44 up-regulated under low iron conditions [38, 39]	**B**
67	BPSS0300 (*malL*)	-	Malonyl coa-acyl carrier protein	– Taurine regulated genes in Bp K96243 [34]<br>– Known as a virulence factor of Bp K96243 [2]<br>– Up-regulated in *bprS* mutant (a sensor histidine kinase gene of two-component signal transduction system mutant) [33]<br>– Computationally derived virulome shared by Bp and Bm [3]	**B**
68	BPSS0303	-	Diaminopimelate decarboxylase	– Taurine regulated genes in Bp K96243 [34]<br>– Known as a virulence factor of Bp K96243 [2]<br>– Up-regulated in *bprRS* mutant [33]<br>– Computationally derived virulome shared by Bp and Bm [3]	**B**
69	BPSS0311	-	Multifunctional polyketide-peptide syntase	– Taurine regulated genes in Bp K96243 [34]<br>– Known as a virulence factor of Bp K96243 [2]<br>– Identified as an attenuated Bp mutant by TraDIS assay [7]<br>– Express in lungs of BALBc [22]<br>– Up-regulated in *bprRS* mutant [33]<br>– Computationally derived virulome shared by Bp and Bm [3]	**B**
70	BPSS0523	+	Hypothetical protein	– Taurine regulated genes in Bp K96243 [34]	**B**

No.	***Sanger ID (Gene name)***	***Strand***	***Product***	***Published finding***	***Group***[#]
				– Computationally derived virulome shared by Bp and Bm [3]	
71	BPSS0524	+	Hypothetical protein	– Taurine regulated genes in Bp K96243 [34]<br>– Predicted as an essential gene for *in vitro* growth of Bp K96243 [4]<br>– *BPSS0529 up-regulated during intracellular growth in U937 [24]*<br>– *BPSS0530 identified as a seroreactive antigen [15] and induced adaptive immunity during acute melioidosis in survivors compared to fatal cases [40]*<br>– Computationally derived virulome shared by Bp and Bm [3]	**B**
72	BPSS1350	-	Efflux/sugar transport/multidrug resistance protein	– Predicted as an essential gene for *in vitro* growth [4]<br>– Taurine regulated genes in Bp K96243 [34]	**B**
73	BPSS2326	-	Flavin-binding monooxygenase-like protein	– Cluster of BPSS2324-29 is taurine regulated gene in Bp K96243 [34]<br>– Computationally derived virulome shared by Bp and Bm [3]	**A**
Transporter genes					
74	BPSL0676	+	Putative transmembrane transporter protein	None	**B**
75	BPSL0922	+	Putative ABC transporter ATP-binding protein	None	**A**
76	BPSL1809	-	Putative amino acid transport system, exported protein	– BPSL1807-09 is an ABC system present in Bp but absent in Bm [41]	**B**
77	BPSL1824	-	Putative ABC transport system, substrate-binding protein	– Involved in core metabolic pathways in Bp but do not have a clear homolog in Bt [42]	**B**
78	BPSL2502	-	ABC transporter, membrane permease	None	**B**
79	BPSS0126	+	Transport system, membrane protein	None	**B**

No.	***Sanger ID (Gene name)***	***Strand***	***Product***	***Published finding***	***Group#***
80	BPSS0293 (*bpeF*)	+	Multidrug-efflux transporter protein	None	**B**
81	BPSS0579 (*braG*)	+	High-affinity branched-chain amino acid transport ATP-binding protein	None	**B**
82	BPSS0951	-	ABC transporter system, ATP-binding protein	None	**A**
83	BPSS1566	+	Probable phosphate transporter	None	**B**
84	BPSS2259	-	Fusion protein, ATP-binding transmembrane ABC transporter and regulatory protein	None	**B**
Recombination and repair genes					
85	BPSL2897 (*ruvC*)	+	Holliday junction resolvase	– *BPSL2898-99 predicted as an essential gene for in vitro growth of Bp K96243*[4]	**B**
Metabolic genes					
86	BPSL0074 (*dnaN*)	-	DNA polymerase III subunit beta	– Predicted as an essential gene for *in vitro* growth [4]<br>– BPSL0073 – 75 constitutively expressed [22]	**B**
87	**BPSL0346***	-	Dihydrodipicolinate synthetase family protein	None	**A**
88	BPSL0542	+	NUDIX domain family protein	None	**B**
89	BPSL1189	-	Putative kinase	None	**B**
90	BPSL1647	+	Putative betaine aldehyde dehydrogenase	– *BPSL1649 – 55 predicted as an essential gene for in vitro growth [4]*	**B**
91	BPSL2243 (*cysS*)	-	Cysteinyl-trna synthetase	– Predicted as an essential gene for *in vitro* growth of Bp K96243 [4]	**B**
92	BPSL2473	-	Putative thymidylate synthase	– Predicted as an essential gene for *in vitro* growth of Bp K96243 [4]	**B**

No.	Sanger ID (Gene name)	Strand	Product	Published finding	Group[#]
93	BPSL2511 (*udg*)	-	Putative UDP-glucose dehydrogenase	– BPSL2509-11 predicted as an essential gene for *in vitro* growth [4]	**B**
94	BPSL2659 (*ureC*)	+	Urease alpha subunit	None	**B**
95	BPSL2843 (*glcD*)	+	Putative glycolate oxidase subunit	None	**B**
96	BPSL3342	-	Putative bacteriophage protein	None	**B**
97	BPSS0130	+	Peptide synthase protein	– Down-regulated in Bp KHW, compared to *bsaN* mutant [17]	**B**
98	BPSS0288	+	Lipoprotein	None	**B**
99	BPSS0339	-	Amino acid dioxygenase	– Down-regulated in Bp KHW, compared to *bsaN* mutant [17]	**B**
100	BPSS0343	-	Aminotransferase protein	None	**A**
101	BPSS0424 (*wbaR*)	+	Glycosyl transferase	None	**B**
102	**BPSS0479***	+	Ribonucleotide reductase protein	None	**A**
103	BPSS0694 (*hpcC*)	+	5-carboxymethyl-2-hydroxymuconate semialdehyde dehydrogenase	– Up-regulated in *bprRS* mutant [33]	**B**
104	BPSS1715 (*gltA*)	-	Citrate synthase	– Predicted as an essential gene for *in vitro* growth [4]<br>– Recruited to Bp expressing BimA (10276 pBHR-virAG) [13]	**B**
105	BPSS1717 (*sdhB*)	-	Succinate dehydrogenase catalytic subunit	– Down-regulated genes under iron-limiting conditions [39]	**B**
106	BPSS1770 (*polA*)	-	DNA polymerase I	None	**B**
107	BPSS2039	+	Cyclopropane-fatty-acyl-phospholipid synthase	– Down-regulated in Bp KHW, compared to *bsaN* mutant, a regulatory gene of T3SS3 and T6SS1 loci is deleted [17]	**A**
108	BPSS2200 (*tyrB2*)	+	Aspartate aminotransferase	– Frameshift mutation cause a virulence strain, MSHR1043 [43]	**B**

No.	Sanger ID (Gene name)	Strand	Product	Published finding	Group[#]
Genes encoding components (Secretion system)					
109	BPSL3101 (*clpB*)	-	Protease associated ATPase	None	**B**
Genetic information processing genes					
110	BPSL1028 (*tnpA*)	-	Transposase	None	**B**
111	BPSL1932 (*tnpA*)	+	Transposase	– Predicted as an essential gene for *in vitro* growth [4]	**B**
112	BPSL2488 (*tnpA*)	-	Transposase	– Predicted as an essential gene for *in vitro* growth [4]	**B**
113	BPSL2817 (*tnpA*)	-	Transposase	None	**B**
114	BPSL3180	-	Putative cytochrome C biogenesis protein	– Predicted as an essential gene for *in vitro* growth of Bp K96243[4]	**B**
115	BPSS1912 (*tnpA*)	-	IS1001 transposase	None	**B**
Regulatory genes					
116	BPSL1130	-	Putative sigma factor	None	**B**
117	BPSL3423	-	Putative Asnc-family transcriptional regulator	None	**B**
118	BPSS0705	+	Response regulator protein	None	**B**
119	BPSS1386	-	ATP/GTP binding protein	None	**B**
120	BPSS2218 (*rpoN2*)	-	DNA-directed RNA polymerase subunit N	None	**B**
Unknown function genes					
121	BPSL1571	-	Hypothetical protein	None	**B**
122	BPSL2274	-	Hypothetical protein	None	**B**
123	BPSL2512	-	Hypothetical protein	– Predicted as an essential gene for *in vitro* growth [4]	**B**
124	BPSL2552	-	Putative lipoprotein	None	**B**

No.	***Sanger ID (Gene name)***	***Strand***	***Product***	***Published finding***	***Group#***
125	BPSL3349	-	Hypothetical protein	– Expression of the operon was not detected when exposed to more than 80 diverse physical, chemical, and biological conditions [22].	**B**
126	BPSS0007	-	Hypothetical protein	None	**A**
127	BPSS0228	-	Hypothetical protein	None	**B**
128	BPSS0504	-	Hypothetical protein	None	**A**
129	BPSS0511	-	Hypothetical protein	None	**B**
130	BPSS2192	+	Hypothetical protein	None	**A**
131	BPSS1014	-	Hypothetical protein	None	**B**
132	BPSS1038	+	Hypothetical protein	– Up-regulated in *bprR* mutant [33]	**B**
133	BPSS1048	+	Hypothetical bacteriophage protein	None	**B**
134	BPSS1082	+	Bacteriophage protein gp17	– Predicted as an essential gene for *in vitro* growth [4]	**B**
135	BPSS1333	+	Hypothetical protein	None	**B**
136	BPSS1820	-	Hypothetical protein	None	**B**
137	BPSS1836	-	Hypothetical protein	None	**A**
138	BPSS2287	+	Hypothetical protein	– Down-regulated under oxidative stress [1]	**B**

Key:

# The start of the mapped regions of the identified genes was upstream of the translational start site or resided within the CDS were assigned to Group A and Group B, respectively.

*** Genes selected for qRT-PCR validation.**

****Genes are selected for mutagenesis.**

Italic: Findings on the adjacent gene that is predicted to be in the same operon of the gene identified downstream of the inducible promoter.

Reference:

1. Jitprasutwit, S., et al., *Transcriptional profiles of Burkholderia pseudomallei reveal the direct and indirect roles of Sigma E under oxidative stress conditions.* BMC Genomics, 2014. **15**: p. 787.
2. Stone, J.K., et al., *Melioidosis: molecular aspects of pathogenesis.* Expert Rev Anti Infect Ther, 2014. **12**(12): p. 1487-99.
3. Schell, M.A., L. Lipscomb, and D. DeShazer, *Comparative genomics and an insect model rapidly identify novel virulence genes of Burkholderia mallei.* J Bacteriol, 2008. **190**(7): p. 2306-13.
4. Moule, M.G., et al., *Genome-wide saturation mutagenesis of Burkholderia pseudomallei K96243 predicts essential genes and novel targets for antimicrobial development.* MBio, 2014. **5**(1): p. e00926-13.
5. Chutoam, P., et al., *RpoS and oxidative stress conditions regulate succinyl-CoA: 3-ketoacid-coenzyme A transferase (SCOT) expression in Burkholderia pseudomallei.* Microbiol Immunol, 2013. **57**(9): p. 605-15.
6. Loprasert, S., et al., *Regulation of the katG-dpsA operon and the importance of KatG in survival of Burkholderia pseudomallei exposed to oxidative stress.* FEBS Lett, 2003. **542**(1-3): p. 17-21.
7. Moule, M.G., et al., *Characterization of New Virulence Factors Involved in the Intracellular Growth and Survival of Burkholderia pseudomallei.* Infect Immun, 2015. **84**(3): p. 701-10.
8. Somprasong, N., et al., *Pseudomonas aeruginosa thiol peroxidase protects against hydrogen peroxide toxicity and displays atypical patterns of gene regulation.* J Bacteriol, 2012. **194**(15): p. 3904-12.
9. Wagley, S., et al., *The twin arginine translocation system is essential for aerobic growth and full virulence of Burkholderia thailandensis.* J Bacteriol, 2014. **196**(2): p. 407-16.
10. Breitbach, K., J. Kohler, and I. Steinmetz, *Induction of protective immunity against Burkholderia pseudomallei using attenuated mutants with defects in the intracellular life cycle.* Trans R Soc Trop Med Hyg, 2008. **102 Suppl 1**: p. S89-94.
11. Pilatz, S., et al., *Identification of Burkholderia pseudomallei genes required for the intracellular life cycle and in vivo virulence.* Infect Immun, 2006. **74**(6): p. 3576-86.
12. Hamad, M.A., et al., *Adaptation and antibiotic tolerance of anaerobic Burkholderia pseudomallei.* Antimicrob Agents Chemother, 2011. **55**(7): p. 3313-23.
13. Jitprasutwit, N., et al., *Identification of Candidate Host Cell Factors Required for Actin-Based Motility of Burkholderia pseudomallei.* J Proteome Res, 2016. **15**(12): p. 4675-4685.
14. Tuanyok, A., et al., *Genome-wide expression analysis of Burkholderia pseudomallei infection in a hamster model of acute melioidosis.* Infect Immun, 2006. **74**(10): p. 5465-76.
15. Felgner, P.L., et al., *A Burkholderia pseudomallei protein microarray reveals serodiagnostic and cross-reactive antigens.* Proc Natl Acad Sci U S A, 2009. **106**(32): p. 13499-504.
16. Lazar Adler, N.R., et al., *Identification of a predicted trimeric autotransporter adhesin required for biofilm formation of Burkholderia pseudomallei.* PLoS One, 2013. **8**(11): p. e79461.

17. Chen, Y., et al., *Characterization and analysis of the Burkholderia pseudomallei BsaN virulence regulon.* BMC Microbiol, 2014. **14**: p. 206.
18. Duong, L.T., et al., *GvmR - A Novel LysR-Type Transcriptional Regulator Involved in Virulence and Primary and Secondary Metabolism of Burkholderia pseudomallei.* Front Microbiol, 2018. **9**: p. 935.
19. Su, Y.C., et al., *A genome level survey of Burkholderia pseudomallei immunome expressed during human infection.* Microbes Infect, 2008. **10**(12-13): p. 1335-45.
20. Erskine, P.T., et al., *High resolution structure of BipD: an invasion protein associated with the type III secretion system of Burkholderia pseudomallei.* J Mol Biol, 2006. **363**(1): p. 125-36.
21. Cuccui, J., et al., *Development of signature-tagged mutagenesis in Burkholderia pseudomallei to identify genes important in survival and pathogenesis.* Infect Immun, 2007. **75**(3): p. 1186-95.
22. Ooi, W.F., et al., *The condition-dependent transcriptional landscape of Burkholderia pseudomallei.* PLoS Genet, 2013. **9**(9): p. e1003795.
23. Burtnick, M.N., et al., *The cluster 1 type VI secretion system is a major virulence determinant in Burkholderia pseudomallei.* Infect Immun, 2011. **79**(4): p. 1512-25.
24. Chieng, S., L. Carreto, and S. Nathan, *Burkholderia pseudomallei transcriptional adaptation in macrophages.* BMC Genomics, 2012. **13**: p. 328.
25. Lazar Adler, N.R., et al., *Systematic mutagenesis of genes encoding predicted autotransported proteins of Burkholderia pseudomallei identifies factors mediating virulence in mice, net intracellular replication and a novel protein conferring serum resistance.* PLoS One, 2015. **10**(4): p. e0121271.
26. Tiyawisutsri, R., et al., *Burkholderia Hep_Hag autotransporter (BuHA) proteins elicit a strong antibody response during experimental glanders but not human melioidosis.* BMC Microbiol, 2007. **7**: p. 19.
27. Balder, R., et al., *Identification of Burkholderia mallei and Burkholderia pseudomallei adhesins for human respiratory epithelial cells.* BMC Microbiol, 2010. **10**: p. 250.
28. Reckseidler-Zenteno, S.L., et al., *Characterization of the type III capsular polysaccharide produced by Burkholderia pseudomallei.* J Med Microbiol, 2010. **59**(Pt 12): p. 1403-14.
29. Chirakul, S., et al., *Characterization of BPSS1521 (bprD), a regulator of Burkholderia pseudomallei virulence gene expression in the mouse model.* PLoS One, 2014. **9**(8): p. e104313.
30. Treerat, P., et al., *The Burkholderia pseudomallei Proteins BapA and BapC Are Secreted TTSS3 Effectors and BapB Levels Modulate Expression of BopE.* PLoS One, 2015. **10**(12): p. e0143916.
31. Warawa, J. and D.E. Woods, *Type III secretion system cluster 3 is required for maximal virulence of Burkholderia pseudomallei in a hamster infection model.* FEMS Microbiol Lett, 2005. **242**(1): p. 101-8.

32. Chin, C.Y., et al., *Global transcriptional analysis of Burkholderia pseudomallei high and low biofilm producers reveals insights into biofilm production and virulence.* BMC Genomics, 2015. **16**: p. 471.
33. Lazar Adler, N.R., et al., *Perturbation of the two-component signal transduction system, BprRS, results in attenuated virulence and motility defects in Burkholderia pseudomallei.* BMC Genomics, 2016. **17**: p. 331.
34. Nandi, T., et al., *A genomic survey of positive selection in Burkholderia pseudomallei provides insights into the evolution of accidental virulence.* PLoS Pathog, 2010. **6**(4): p. e1000845.
35. Suwannasaen, D., et al., *Human immune responses to Burkholderia pseudomallei characterized by protein microarray analysis.* J Infect Dis, 2011. **203**(7): p. 1002-11.
36. Chua, K.L., Y.Y. Chan, and Y.H. Gan, *Flagella are virulence determinants of Burkholderia pseudomallei.* Infect Immun, 2003. **71**(4): p. 1622-9.
37. Eng, S.A. and S. Nathan, *Curcumin rescues Caenorhabditis elegans from a Burkholderia pseudomallei infection.* Front Microbiol, 2015. **6**: p. 290.
38. Tuanyok, A., et al., *Genome-wide expression analysis of iron regulation in Burkholderia pseudomallei and Burkholderia mallei using DNA microarrays.* FEMS Microbiol Lett, 2005. **252**(2): p. 327-35.
39. Alice, A.F., et al., *Genetic and transcriptional analysis of the siderophore malleobactin biosynthesis and transport genes in the human pathogen Burkholderia pseudomallei K96243.* J Bacteriol, 2006. **188**(4): p. 1551-66.
40. Dunachie, S.J., et al., *Infection with Burkholderia pseudomallei - immune correlates of survival in acute melioidosis.* Sci Rep, 2017. **7**(1): p. 12143.
41. Harland, D.N., et al., *ATP-binding cassette systems in Burkholderia pseudomallei and Burkholderia mallei.* BMC Genomics, 2007. **8**: p. 83.
42. Yu, Y., et al., *Genomic patterns of pathogen evolution revealed by comparison of Burkholderia pseudomallei, the causative agent of melioidosis, to avirulent Burkholderia thailandensis.* BMC Microbiol, 2006. **6**: p. 46.
43. Price, E.P., et al., *Within-host evolution of Burkholderia pseudomallei over a twelve-year chronic carriage infection.* MBio, 2013. **4**(4).
